# Azaacene Dimers: Acceptor Materials with a Twist

**DOI:** 10.1002/chem.201904683

**Published:** 2019-12-19

**Authors:** Lukas Ahrens, Julian Butscher, Victor Brosius, Frank Rominger, Jan Freudenberg, Yana Vaynzof, Uwe H. F. Bunz

**Affiliations:** ^1^ Organisch Chemisches Institut Ruprecht-Karls-Universität Heidelberg Im Neuenheimer Feld 270 69120 Heidelberg Germany; ^2^ Centre for Advanced Materials (CAM) and Kirchhoff Institute for Physics Im Neuenheimer Feld 225 & 227 69120 Heidelberg Germany

**Keywords:** azaacenes, electron acceptors, organic photovoltaics, semiconductors, spiro compounds

## Abstract

The synthesis of five spiro‐linked azaacene dimers is reported and their properties are compared to that of their monomers. Dimerization quenches emission of the longer (≥(hetero)tetracenes) derivatives and furnishes amorphous thin‐films, the absorption is not affected. The larger derivatives were tested as acceptors in bulk‐heterojunction photovoltaic devices with a maximum power conversion efficiency of up to 1.6 %.

## Graphical abstract

**Let's twist again**: Simple condensation reactions with a spiro‐fused bis‐quinone furnishes amorphous azaacene dimers applicable as acceptors in organic photovoltaics (see graphic).

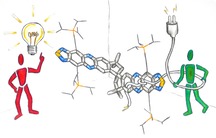

Acenes^1^ and their N‐heterocyclic counterparts^2^ are indispensable organic semiconductors with applications that range from thin‐film transistors,2d, 3 organic light‐emitting diodes^4^ to organic photovoltaic devices (OPV).^5^ Both acenes and azaacenes display additional physical processes of interest, such as singlet fission (SF).^6^, ^7^ Intermolecular SF relies on the close proximity of two (or potentially more) acene or azaacene units in the bulk/thin‐films or concentrated solutions. Well‐defined molecular dimers allow a transfer to dilute systems, rendering SF intramolecular by weak coupling of the acene partners and independent of morphological effects or collisions. Campos et al. investigated rigid homoconjugated pentacenes in which a spiro‐bridge is incorporated for intramolecular SF (Figure 1).^8^

**Figure 1:**
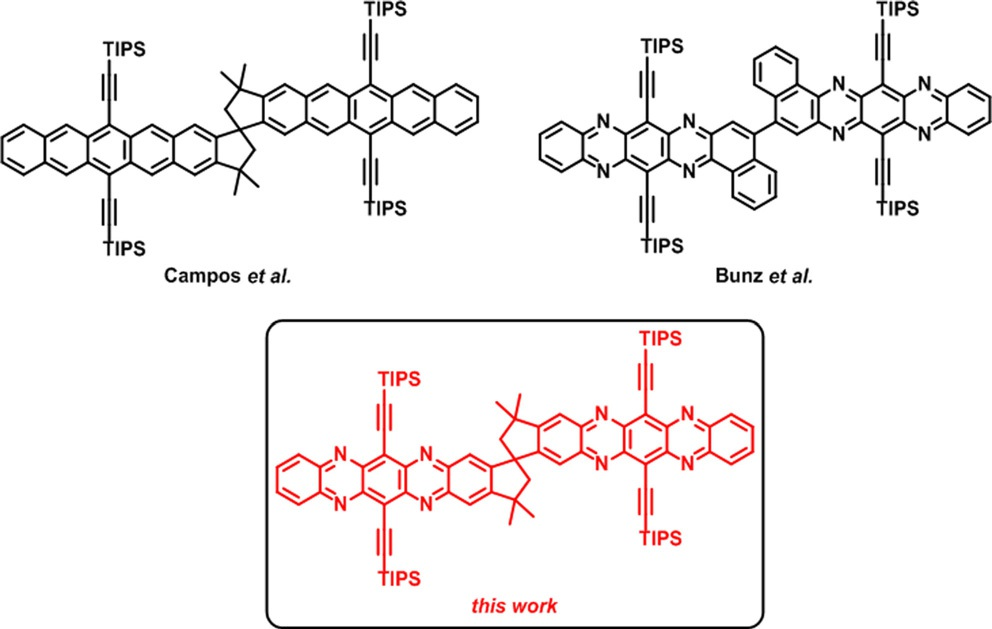
Selected examples of (aza)arene dimers and leitmotif investigated herein.

Connecting arenes using **1** gives rigid polymers of intrinsic microporosity,^9^ which, although being soluble, are porous due to nonefficient packing of the polymer strands.

Azaacenes are tailor‐made and electronically modulated by substitution, yet their strong tendency to π‐stack results in crystalline domains in the solid‐state and has hindered their widespread use as active layer acceptors in bulk‐heterojunction OPV. Balancing acceptor character, charge transport and aggregation/morphology is challenging, but can be resolved for azaacenes either by incorporation of morphology‐dominating units, such as iptycenes4a which also enhance solubility^10^ or even simple oligomer formation,^11^ for example, the azaphene‐dimer shown in Figure 1.5c

We herein disclose spiro‐connected azaacenes that form easily and in high yields. We demonstrate their use as acceptor materials in proof‐of‐concept OPVs. The critical building block is compound **1**, obtained by oxidation (NaIO_4_ in EtOH/DCM, 94 %) of the tetrahydroxy‐species employed by McKeown to prepare porous polymers of intrinsic microporosity.9a

Compound **1** is stable and can be stored without decomposition at ambient temperature. Yet it is highly reactive in condensation reactions with the diamines **2 a**–**e** and furnishes the spiro‐bridged targets **3 a**–**e** in 66 to 86 % yield after flash column chromatography and gel permeation chromatography (Scheme 1). We note that the condensation conditions are mild, 50 °C. Often higher reaction temperatures are required for better conversion.4b, 12

**Scheme 1:**
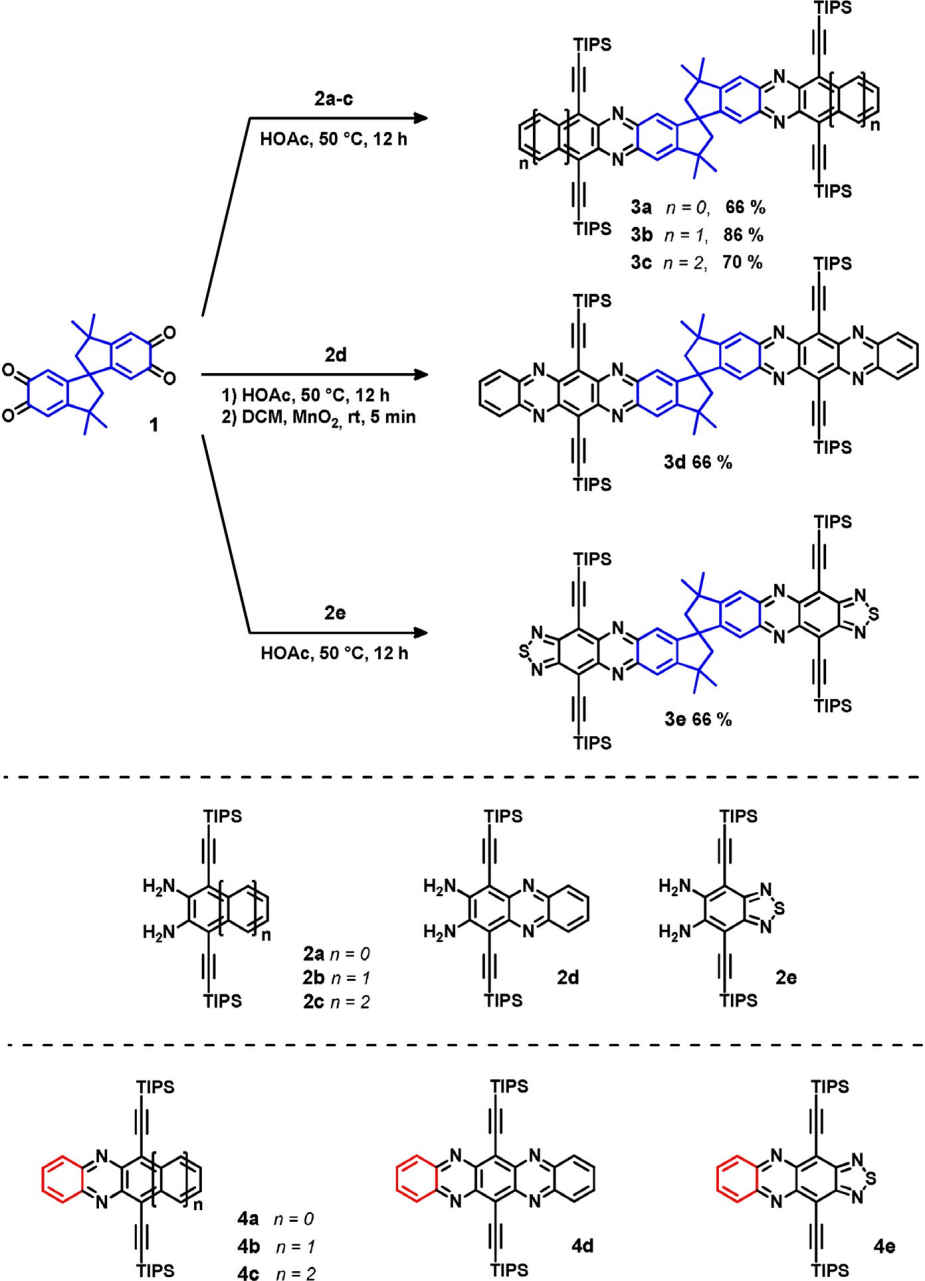
Condensation of *ortho*‐quinone dimer **1** with diamines **2 a**‐**e** (middle) to produce azaacene dimers **3 a**–**e** (top). Bottom: monomeric reference compounds **4 a**–**e**.

Figure 2 displays photographs of dilute solutions (*n*‐hexane) of the spiro‐bridged dimers **3 a**–**e** and their monomers **4 a**–**e** (cf. Scheme 1, bottom) in daylight and under blacklight irradiation (365 nm). With the exception of the azapentacene series **e** the colors of the solutions do not change. Concerning photoluminescence, **4 b** and **3 b** are fairly fluorescent, while in **e** the fluorescence is quenched when going from monomeric **4 e** to dimeric **3 e**, most likely testimony to the excited state interaction between the two acene units.

**Figure 2:**
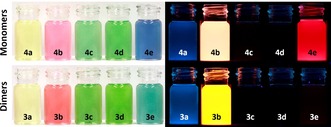
Photographs of monomers **4 a**–**e** (top, from left to right) and dimers **3 a**–**e** (bottom, from left to right) under daylight (left) and UV light with excitation at 365 nm (right) in *n*‐hexane.

Figure 3 displays the UV/Vis spectra in solution and in the solid state; Table 1 summarizes the optical properties of the compounds **3 a**–**e** and their companion molecules **4 a**–**e**. Comparing monomers **4** to the dimer series **3**, the differences of the spectra in solution are minute—for **3 a**–**e** there is a minor redshift in absorption compared to **4 a**–**e**. This observation could be due to the electronic effect that is exerted by the (formal) double alkyl substitution in these electron‐deficient species or, but not necessarily, due to interactions of the backbones via homoconjugation. The spun‐cast thin‐film UV/Vis spectra of the **3** series are well resolved as **3 a**–**e** all form amorphous films. For **3 c**–**e** slight redshifts are observed when going into the solid state, hinting at a small intermolecular coupling of the chromophores. In contrast, monomers **4 a**–**e** show broadened and less resolved thin film spectra: In case of **4 c**–**e**, these effects are strongest, testimony to the electronic interactions between the π‐systems of the acenes in the solid‐state. The spiro‐linker influences morphology: While **4 d** is crystalline, **3 d** is amorphous as investigated by polarizing light microscopy (Figure 4), supporting their differing solid‐state absorptions. Similar observations were made for the remaining heteroacene monomers and dimers.

**Figure 3:**
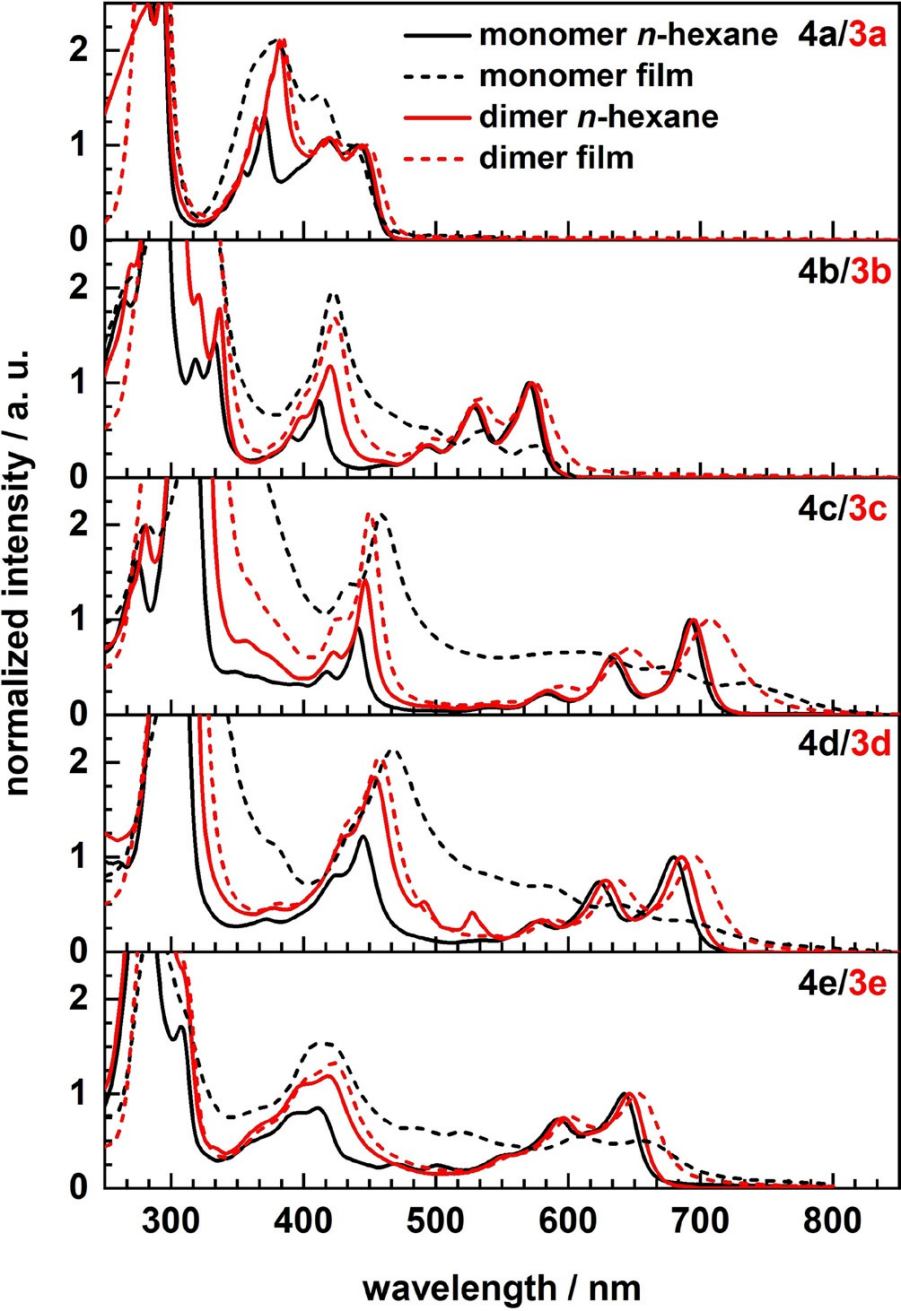
Normalized absorption spectra of monomers **4 a**–**e** (black) and dimers **3 a**–**e** (red) in *n*‐hexane (dilute solution) and spun‐cast thin‐films on glass (dashed, from chloroform; *c*=10 mg mL^−1^).

**Table 1:** Optical properties of monomeric acenes **4 a**–**e** and azaacene dimers **3 a**–**e** in *n*‐hexane.

Compd	*λ*_max, abs_ [nm]	*λ*_onset, abs_ [nm]	*λ*_max, em_ [nm]	Stokes shift [cm^−1^]	*ϵ* [×10^4^ m ^−1^ cm^−1^]^[a]^	*Φ*	*τ* [ns]
**4 a**	440	464	462	1073	–	0.02^[b]^	0.37^[b]^
**3 a**	444	470	464	971	10.1	0.01	0.1
**4 b**	570	592	579	273	2.27^[c]^	0.09^[c]^	–
**3 b**	573	599	583	299	20.3	0.37	12.3
**4 c**	692	714	698	124	1.91^[c]^	<0.01^[c]^	–
**3 c**	695	719	703	164	29.1	–^[d]^	–^[d]^
**4 d**	680	704	688	171	–	–	–
**3 d**	686	714	695	189	23.8	–^[d]^	–^[d]^
**4 e**	642	667	651	215	1.72^[e]^	–	–
**3 e**	646	673	657	259	13.0	–^[d]^	–^[d]^

[a] At *λ*_max, abs_. [b] Data taken from ref. 21 in DCM. [c] Data taken from ref. 22. [d] Nonfluorescent. [e] Data taken from ref. 16.

**Figure 4:**
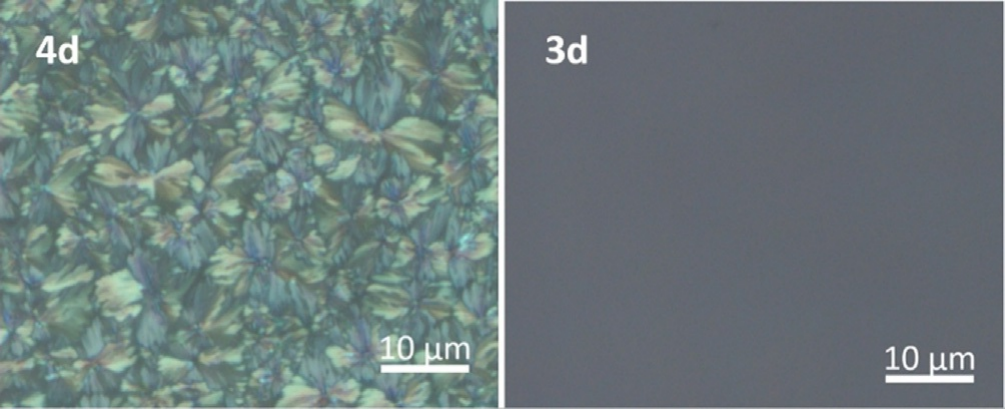
Microscopic images of spin‐coated thin films of **4 d** and **3 d** on glass (chloroform, 10 mg mL^−1^) under crossed polarizers.

Table 2 displays the general electronic properties of **3 a**–**e** and **4 a**–**e** as obtained by cyclic voltammetry and by quantum‐chemical calculations. In general, the calculated values for the HOMOs are 0.1 eV higher for dimers **3** than those of **4**, testament of the electron donating cyclopentane ring attached to the azaacene core. The LUMO positions are similarly affected by 0.07 eV. Redox potentials are in reasonable agreement but can display a deviation of up to 0.2 eV from the calculated electron affinities/ionization potentials—not unexpected when comparing values from different sources and methods. Overall, the electronic properties in the ground state are not strongly affected when going from **4** to the spiro compounds **3**.

**Table 2:** Experimental and calculated (gas‐phase) properties of monomeric acenes **4 a**–**e** and azaacene dimers **3 a**–**e** in solution (*n*‐hexane).

Compd.	*E*_(0/−)_	Ionization potential/HOMO [eV]^[d]^	Electron affinity/LUMO [eV]^[b]^	gap [eV]^[c]^
	[V]^[a]^	meas/^[e]^calcd	meas/^[e]^calcd	meas/^[e]^calcd
**4 a** ^[f]^	−1.68	−5.82/−5.97	−3.12/−3.08	2.70/2.88
**3 a**	−1.68	−6.16/−5.87	−3.52/−3.01	2.64/2.86
**4 b** ^[f]^	−1.23	−5.69/−5.54	−3.57/−3.35	2.12/2.20
**3 b**	−1.03	−5.87/−5.44	−3.80/−3.27	2.07/2.17
**4 c** ^[f]^	−1.05	−5.50/−5.25	−3.75/−3.50	1.75/1.75
**3 c**	−1.20	−5.65/−5.15	−3.93/−3.43	1.72/1.72
**4 d** ^[f]^	−0.79	−5.83/−5.60	−4.01/−3.84	1.82/1.76
**3 d**	−1.05	−5.95/−5.49	−4.21/−3.79	1.74/1.70
**4 e** ^[f]^	−0.83	−5.83/−5.76	−3.97/−3.87	1.86/1.89
**3 e**	−0.83	−5.99/−5.67	−4.15/−3.80	1.84/1.87

[a] First reduction potentials from cyclic voltammetry (CV) in DCM at room temperature with Bu_4_NPF_6_ as the electrolyte against Fc/Fc^+^ as an internal standard (−5.10 eV) at 0.2 V s^−1^.^13^ [b] Electron affinity_meas_=−e×(5.1 V+*E*_Red._). [c] gap_meas_ calculated from *λ*_onset_ in *n*‐hexane. [d] Ionization potential_meas_=electron affinity_meas_−gap_meas_. [e] Obtained from DFT calculations (TURBOMOLE B3LYP/def2‐TZVP// Gaussian 09 B3LYP/6‐311++G**; TMS groups were used instead of TIPS). [f] Data for **4 a**–**c** (THF) were taken from ref. 14, for **4 d** (DCM) from ref. 15 and for **4 e** (THF) from ref. 16.

We obtained a single‐crystalline specimen of **1** (Figure 5 a) and note that **1** crystallizes as a racemate. Both enantiomers *aR* and *aS* are identified by the single‐crystal structure analysis. Consequently, all the target compounds **3 a**–**e** must also form as racemic mixtures. The small derivatives **3 a,b** are amorphous, but we obtained specimen of **3 c** and **3 e** suitable for X‐ray crystallography (Figure 5 b–g), which show similar molecular packing: They form chains, in which *aS* and *aR* enantiomers alternate. For **3 c**, neighboring molecules π‐stack weakly via the acene backbones—note that obtaining a suitable crystal, even though only for a qualitative discussion, took various attempts. In the case of **3 e**, the molecular packing is dominated by intermolecular S,N‐interactions. Both unit cells of **3 c** and **3 e** and their reluctance to form crystalline domains explain the small redshift in thin‐films—significant π–π‐interactions are not observed.

**Figure 5:**
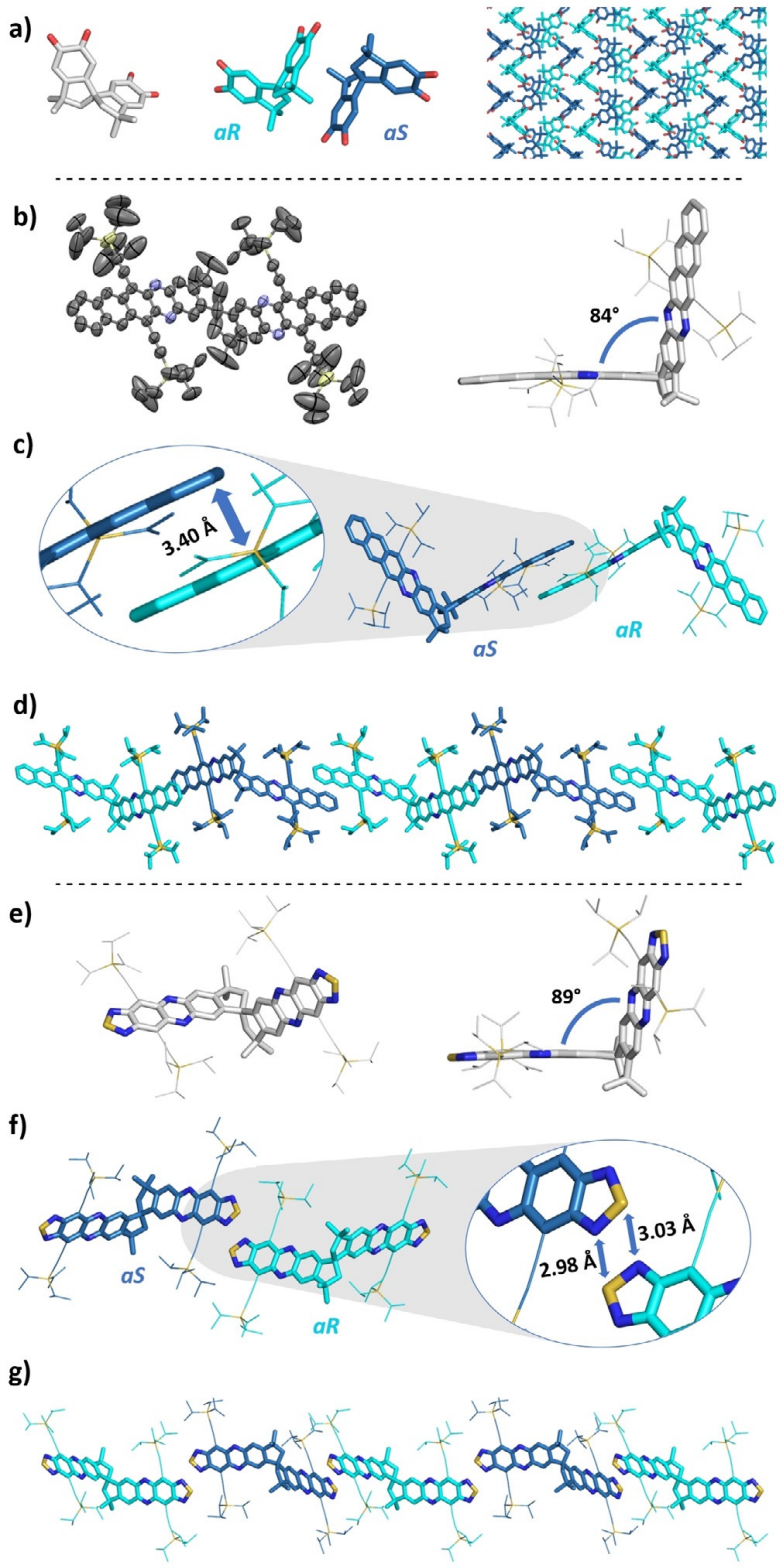
Solid‐state structures of **1** (a), **3 c** (b) and **3 e** (e) including twist angle between acene subunits; π‐stacking in **3 c** (c) and S‐N short contacts in **3 e** (f), visualization of packing (d,g); enantiomers are highlighted by different colors.

Compounds **3 c**–**e** were investigated as acceptors in organic bulk heterojunction (BHJ) solar cells employing an inverted architecture as depicted in Figure 6 a. The organic polymer poly({4,8‐bis[(2‐ethylhexyl)oxy]benzo[1,2‐*b*:4,5‐*b′*]dithio‐phene‐2,6‐diyl}{3‐fluoro‐2‐[(2‐ethylhexyl)carbonyl]thieno[3,4‐*b*]thio‐phenediyl}) (**PTB7**) was used as a donor in all devices. Figure 6 c shows the external quantum efficiency for devices with each acceptor compound. The features below 500 nm indicate that the acceptor contributes to the photocurrent generation since the donor **PTB7** absorbs far more weakly in this spectral range. In Figure 6 d the *J*–*V* curves for the optimal devices of **PTB7** with each of the acceptor molecules are depicted. As expected from the external quantum efficiency spectra, the short‐circuit current increases from compound **3 c** to **3 e**. On the other hand, the open‐circuit voltage (*V*_OC_) of the **3 c** device is much higher than that of devices with **3 d** or **3 e**. This increase in *V*_OC_ stems from a much larger photovoltaic gap (cf. Table 2) as a result of the lower electron affinity of **3 c** compared to **3 d,e**. All devices exhibit a low fill factor, which we attribute to an unfavorable morphology of the BHJ active layer. Interestingly, the **3 e** derivative results in the highest performance, similar to previous observations for other phenazino‐thiadiazole derivatives.^5^ Finally, Table 3 summarizes the average and maximum performance of a total of 44 devices, confirming the trend discussed for the optimal devices.

**Figure 6:**
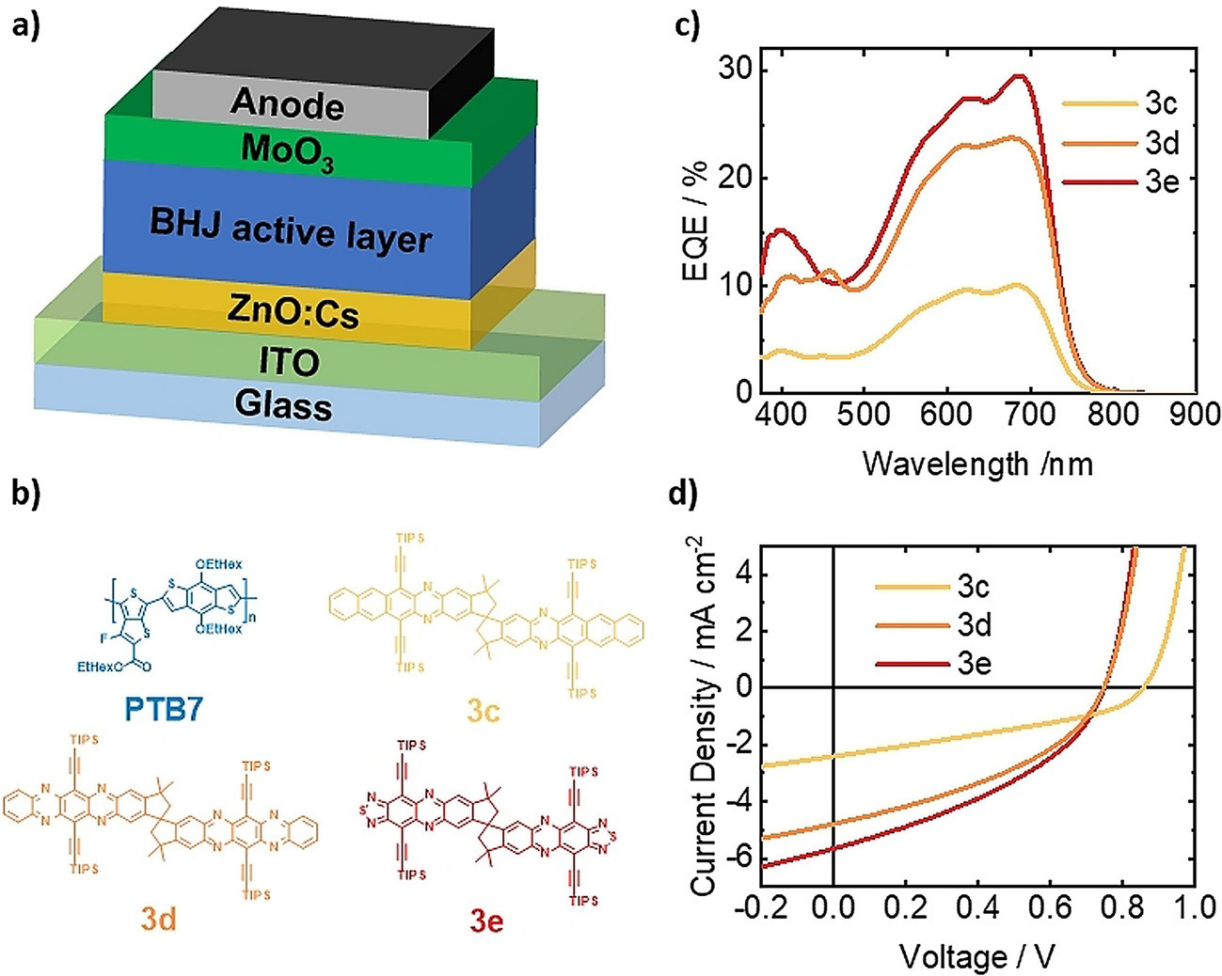
Schematic representation of photovoltaic device structure (a) and chemical structures of donor **PTB7** poly({4,8‐bis[(2‐ethylhexyl)oxy]benzo[1,2‐*b*:4,5‐*b*′]dithiophene‐2,6‐diyl}{3‐fluoro‐2‐[(2‐ethylhexyl)carbonyl]thieno[3,4‐*b*]‐thiophenediyl} and acceptor materials **3 c–e** (b). External quantum efficiency of devices with different acceptor materials (c) and current–voltage characteristics of optimal devices (d).

**Table 3:** Photovoltaic parameter statistics of solar cells fabricated by using **3 c**–**e** as acceptors with **PTB7** as the donor. The values in brackets denote the best performance obtained for each derivative.

Acceptor	*V*_OC_^[a]^ [V]	*J*_SC_^[a]^ [mA cm^−2^]	FF^[a]^ [%]	PCE^[a]^ [%]
**3 c**	0.85±0.01	−2.24±0.08	34.9±0.4	0.66±0.03
	(0.86)	(−2.40)	(35.6)	(0.733)
**3 d**	0.73±0.01	−4.47±0.16	38.1±1.0	1.25±0.08
	(0.75)	(−4.79)	(39.5)	(1.41)
**3 e**	0.74±0.01	−5.38±0.12	37.7±0.7	1.50±0.06
	(0.75)	(−5.66)	(38.3)	(1.62)

[a] *V*_OC_=open‐circuit voltage; *J*_SC_=short‐circuit current; FF=fill factor; PCE=power conversion efficiency.

In conclusion, we have prepared spiro‐linked azaacene dimers **3 a**–**e** and compared them to their consanguine monomers **4 a**–**e**. Fusion of the azaacene subunits onto the rigid spiro‐scaffold leads to a quenched emission of the longer derivative **3 e** and significantly alters thin‐film morphology, furnishing amorphous films. Absorption, however, remains unaltered, even in spun‐cast films. Electron‐accepting **3 c**–**e** were investigated as acceptors in BHJ photovoltaic devices yielding—without extensive optimization—a maximum power conversion efficiency of about 1.6 %. Our results demonstrate the potential of the examined compounds as acceptors in BHJ solar cells. Investigation of intramolecular singlet fission of our larger dimers, also with respect to control of the twisting angle, is underway.

 

 

 

## Experimental Section

### Syntheses

Compounds **2 a–c** 17, 18, 19, 20 and **4 a–d**,^15^, ^18^, ^21^, ^22^ were synthesized according to literature procedure.

CCDC https://www.ccdc.cam.ac.uk/services/strctures?id=doi:10.1002/chem.201904683 (**1**, **3 c**, **3 e**, respectively) contain the supplementary crystallographic data for this paper. These data are provided free of charge by http://www.ccdc.cam.ac.uk/.

### General procedure for the preparation of azaacene dimers

In a heatgun dried Schlenk tube under an atmosphere of argon was added quinone **1** (1.0 equiv) and the *ortho*‐diamine **2** (2.0 equiv). Then chloroform and acetic acid were added and the reaction mixture was stirred at 50 °C for 12 h. The mixture was cooled to room temperature and diluted with water (10 mL). The phases were separated and the aqueous layer was extracted with dichloromethane (3×10 mL). The combined organic phases were washed with sodium bicarbonate solution (10 mL), dried over magnesium sulfate and filtrated. The solvent was removed under reduced pressure and the crude product was absorbed on Celite^®^. After flash column chromatography (petroleum ether/diethyl ether 250:1 v/v) and gel permeation chromatography (toluene) the product **3** was isolated.

### 3,3,3′,3′‐Tetramethyl‐2,2′,3,3′‐tetrahydro‐1,1′‐spirobi[1*H*‐indene]‐5,5′,6,6′‐tetraone (1)

In a flask was dissolved racemic 3,3,3′,3′‐tetramethyl‐2,2′,3,3′‐tetrahydro‐1,1′‐spirobi[1*H*‐indene]‐5,5′,6,6′‐tetraol (3.00 g, 8.81 mmol, 1.00 equiv) in 40 mL ethanol, which was then diluted with 150 mL dichloromethane. A solution of sodium periodate (4.15 g, 19.4 mmol, 2.20 equiv) in 40 mL distilled water was added and the reaction mixture stirred at room temperature for 1 h. The phases were separated and the aqueous layer was extracted with dichloromethane (3×30 mL). The combined organic phases were washed with brine (20 mL), dried over magnesium sulfate and filtrated. The solvent was removed under reduced pressure and the title compound **1** was isolated as a red solid (2.78 g, 8.28 mmol, 94 %). ^1^H NMR (CDCl_3_, 301 MHz, rt): *δ*=6.27 (d, *J=*0.55 Hz, 2 H), 6.17 (d, *J=*0.55 Hz, 2 H), 2.38 (d, *J=*13.5 Hz, 2 H), 2.24 (d, *J=*13.5 Hz, 2 H), 1.41 (s, 6 H), 1.39 (s, 6 H) ppm. All analytical data is in good agreement with literature.^23^ A single‐crystalline specimen was obtained by slow diffusion of methanol into a chloroform solution of **1**.

### 4,11‐Bis[(triisopropylsilyl)ethynyl]‐[1,2,5]thiadiazolo[3,4‐*b*]phenazine (4 e)

In a heatgun dried Schlenk tube under an atmosphere of argon was suspended **2 d** (100 mg, 175 μmol, 1.00 equiv) in 1.5 mL anhydrous pyridine. *N*‐Thionylaniline (41.3 μL, 51.2 mg, 368 μmol, 2.10 equiv) was added and after stirring the reaction mixture at 80 °C for 5 min, trimethylsilyl chloride (222 μL, 190 mg, 1.75 mmol, 10.0 equiv) was slowly added to the suspension. The reaction mixture was stirred at 80 °C for 6 h. The mixture was cooled to room temperature and diluted with 10 mL DCM. The mixture was washed with 1 n hydrochloric acid (2×10 mL) and sodium bicarbonate solution (10 mL), dried over magnesium sulfate and filtrated. The solvent was removed under reduced pressure and the crude product was absorbed on Celite^®^. After flash column chromatography (petroleum ether/diethyl ether 250:1 *v*/*v*→100:1→50:1) and gel permeation chromatography (toluene) the product **4 e** was isolated as a dark‐blue solid (21.6 mg, 36.1 μmol, 21 %). ^1^H NMR (CDCl_3_, 301 MHz, rt): *δ*=8.09–8.16 (m, 2 H), 7.75–7.82 (m, 2 H), 1.30–1.34 ppm (m, 42 H); All analytical data is in good agreement with literature.^16^

### 3,3,3′,3′‐Tetramethyl‐6,6′,9,9′‐tetrakis[(triisopropylsilyl)ethynyl]‐2,2′,3,3′‐tetrahydro‐1,1′‐spirobi[cyclopenta[*b*]phenazine] (3 a)

The GP was applied to **1** (100 mg, 297 μmol, 1.00 equiv) and **2 a** (279 mg, 595 μmol, 2.00 equiv) in 1.5 mL chloroform and 1.5 mL acetic acid. Flash column chromatography (SiO_2_; petroleum ether/diethyl ether 250:1 v/v) and gel permeation chromatography (toluene) yielded a yellow solid **3 a** (237 mg, 197 μmol, 66 %). *R*_f_=0.76 (SiO_2_; petroleum ether/dichloromethane 2:1, *v*/*v*). M.p.: 185 °C; ^1^H NMR (CDCl_3_, 600 MHz, rt): *δ*=8.06 (s, 2 H), 7.90 (d, *J=*7.40 Hz, 2 H), 7.86 (d, *J=*7.40 Hz, 2 H), 7.64 (s, 2 H), 2.66–2.73 (m, 4 H), 1.70 (s, 6 H), 1.60 (s, 6 H), 1.27–1.31 (m, 42 H), 1.06–1.11 ppm (m, 42 H); ^13^C NMR (CDCl_3_, 151 MHz, rt): *δ*=158.2, 157.6, 143.8, 143.8, 143.0, 142.9, 133.6, 133.4, 124.9, 124.5, 124.2, 122.0, 104.1, 103.8, 100.8, 100.4, 60.3, 57.4, 44.0, 32.1, 30.6, 19.0, 18.8, 11.7, 11.5 ppm; IR (ATR): $\tilde{\nu }$ =2941, 2863, 1461, 1036, 996, 880, 847, 787, 674, 660, 644, 633, 581, 457 cm^−1^; HRMS (MALDI^+^): *m*/*z*: [*M*]^.+^: calcd for [C_77_H_108_N_4_Si_4_]^.+^: 1200.7646; found: 1200.7693; correct isotope distribution.

### 3,3,3′,3′‐Tetramethyl‐6,6′,11,11′‐tetrakis[(triisopropylsilyl)ethynyl]‐2,2′,3,3′‐tetrahydro‐1,1′‐spirobi[benzo[*b*]cyclopenta[*i*]phenazine] (3 b)

The GP was applied to **1** (40.0 mg, 119 μmol, 1.00 equiv) and **2 b** (123 mg, 238 μmol, 2.00 equiv) in 1.0 mL chloroform and 1.0 mL acetic acid. Flash column chromatography (SiO_2_; petroleum ether/diethyl ether 250:1 *v*/*v*) and gel permeation chromatography (toluene) yielded red solid **3 b** (133 mg, 102 μmol, 86 %). *R*_f_=0.74 (SiO_2_; petroleum ether/dichloromethane 2:1, *v*/*v*); M.p.: 212 °C; ^1^H NMR (CDCl_3_, 600 MHz, rt): *δ*=8.75 (d, *J=*8.08 Hz, 2 H), 8.69 (d, *J=*8.01 Hz, 2 H), 8.06 (s, 2 H), 7.68 (s, 2 H), 7.59–7.67 (m, 4 H), 2.66–2.80 (m, 4 H), 1.74 (s, 6 H), 1.64 (s, 6 H), 1.33–1.44 (m, 42 H), 1.09–1.21 ppm (m, 42 H); ^13^C NMR (CDCl_3_, 126 MHz, rt): *δ*=158.8, 158.2, 144.9, 144.8, 141.1, 140.9, 135.2, 135.0, 127.9, 127.8, 127.7, 127.7, 125.0, 122.0, 120.9, 120.5, 108.2, 107.8, 103.2, 102.9, 60.2, 57.3, 43.9, 32.0, 30.6, 19.1, 18.9, 11.8, 11.6 ppm; IR (ATR): $\tilde{\nu }$ =2941, 2862, 1461, 1442, 1389, 1367, 1042, 995, 879, 759, 675, 660, 593, 482, 403 cm^−1^. HRMS (MALDI^+^): *m*/*z*: [*M*]^.+^: calcd for [C_85_H_112_N_4_Si_4_]^.+^: 1300.7959; found 1300.7983; correct isotope distribution.

### 3,3,3′,3′‐Tetramethyl‐6,6′,13,13′‐tetrakis[(triisopropylsilyl)ethynyl]‐2,2′,3,3′‐tetrahydro‐1,1′‐spirobi[cyclopenta[*b*]naphtho[2,3‐*i*]phena‐zine] (3 c)

The GP was applied to **1** (85.7 mg, 255 μmol, 1.00 equiv) and **2 c** (290 mg, 510 μmol, 2.00 equiv) in 1.5 mL chloroform and 1.5 mL acetic acid. Flash column chromatography (SiO_2_; petroleum ether/diethyl ether 250:1 *v*/*v*) and gel permeation chromatography (toluene) yielded a green solid **3 c** (251 mg, 179 μmol, 70 %). *R*_f_=0.73 (SiO_2_; petroleum ether/dichloromethane 2:1, *v*/*v*). M.p.: ≥400 °C; ^1^H NMR (CDCl_3_, 600 MHz, rt): *δ*=9.42 (s, 2 H), 9.37 (s, 2 H), 8.00 (s, 2 H), 7.97–8.03 (m, 4 H), 7.64 (s, 2 H), 7.43–8.48 (m, 4 H), 2.75 (d, *J=*13.5 Hz, 2 H), 2.70 (d, *J=*13.5 Hz, 2 H), 1.74 (s, 6 H), 1.64 (s, 6 H), 1.40–1.48 (m, 42 H), 1.16–1.26 ppm (m, 42 H); ^13^C NMR (CDCl_3_, 151 MHz, rt): *δ*=159.1, 158.5, 145.3, 145.3, 140.9, 140.7, 133.0, 132.9, 132.7, 132.6, 128.8, 128.8, 127.0, 126.8, 126.8, 126.7, 125.2, 122.1, 120.8, 120.3, 109.6, 109.1, 104.2, 103.8, 60.1, 57.3, 44.0, 32.0, 30.5, 19.2, 19.0, 11.9, 11.7 ppm; IR (ATR): $\tilde{\nu }$ =2940, 2862, 1460, 1375, 1139, 1016, 994, 751, 740, 668, 651, 578, 462, 418 cm^−1^. HRMS (MALDI^+^): *m*/*z*: [*M*]^.+^: calcd for [C_93_H_116_N_4_Si_4_]^.+^: 1400.8272; found: 1400.8243; correct isotope distribution. Single‐crystalline specimen were obtained by slow diffusion of methanol into a chloroform solution of **3 c**.

### 3,3,3′,3′‐Tetramethyl‐6,6′,13,13′‐tetrakis[(triisopropylsilyl)ethynyl]‐2,2′,3,3′‐tetrahydro‐1,1′‐spirobi[cyclopenta[*b*]quinoxalino[2,3‐*i*]phe‐nazine] (3 d)

The GP was applied to **1** (100 mg, 297 μmol, 1.00 equiv) and **2 d** (279 mg, 5.95 μmol, 2.00 equiv) in 1.5 mL chloroform and 1.5 mL acetic acid. Flash column chromatography (SiO_2_; petroleum ether/diethyl ether 250:1 v/v →100:1 →50:1) and gel permeation chromatography (toluene) yielded green solid 3d (277 mg, 197 μmol, 66 %). *R*_f_=0.40 (SiO_2_; petroleum ether/dichloromethane 2:1, *v*/*v*). M.p.: ≥400 °C. ^1^H NMR (CDCl_3_, 500 MHz, rt): *δ*=8.16–8.24 (m, 4 H), 8.07 (s, 2 H), 7.77–7.84 (m, 4 H), 7.70 (s, 2 H), 2.70–2.80 (m, 4 H), 1.76 (s, 6 H), 1.66 (s, 6 H), 1.39–1.44 (m, 42 H), 1.17–1.25 ppm (m, 42 H); ^13^C NMR (CDCl_3_, 126 MHz, rt): *δ*=160.1, 159.4, 145.8, 145.8, 145.5, 145.5, 143.0, 142.9, 142.7, 142.5, 132.0, 132.0, 130.7, 130.6, 125.3, 122.9, 122.5, 122.4, 112.7, 112.1, 103.4, 103.1, 60.0, 57.5, 44.1, 32.0, 30.5, 19.2, 19.0, 11.9, 11.7 ppm; IR (ATR): $\tilde{\nu }$ =2940, 2862, 1452, 1429, 1382, 1311, 1117, 1023, 996, 881, 734, 582, 419 cm^−1^; HRMS (MALDI^+^): *m*/*z*: [*M*+H]^.+^: calcd for [C_89_H_113_N_8_Si_4_]^.+^: 1405.8160; found: 1405.8148; correct isotope distribution.

### 3,3,3′,3′‐Tetramethyl‐6,6′,9,9′‐tetrakis[(triisopropylsilyl)ethynyl]‐2,2′,3,3′‐tetrahydro‐1,1′‐spirobi[cyclopenta[*b*]phenazine] (3e)

The GP was applied to **1** (150 mg, 446 μmol, 1.00 equiv) and **2 e** (470 mg, 892 μmol, 2.00 equiv) in 2.0 mL chloroform and 2.0 mL acetic acid. Flash column chromatography (SiO_2_; petroleum ether/diethyl ether 250:1 *v*/*v* →100:1→50:1) and gel permeation chromatography (toluene) yielded green solid **3 e** (389 mg, 295 μmol, 66 %). *R*_f_=0.42 (SiO_2_; petroleum ether/dichloromethane 2:1, *v*/*v*). M.p.: 325 °C. ^1^H NMR (CDCl_3_, 600 MHz, rt): *δ*=7.96 (s, 2 H), 7.59 (s, 2 H), 2.68–2.75 (m, 4 H), 1.73 (s, 6 H), 1.63 (s, 6 H), 1.33–1.41 (m, 42 H), 1.12–1.21 ppm (m, 42 H); ^13^C NMR (CDCl_3_, 126 MHz, rt): *δ*=160.1, 159.5, 154.8, 154.7, 145.8, 145.7, 142.5, 142.4, 125.0, 122.2, 114.4, 114.1, 111.9, 111.4, 102.3, 102.1, 59.9, 57.3, 44.1, 31.9, 30.4, 19.1, 18.9, 11.8, 11.6 ppm. IR (ATR): $\tilde{\nu }$ =2941, 2862, 1454, 1447, 1364, 1200, 1018, 996, 920, 861, 658, 646, 599, 572 cm^−^; HRMS (MALDI^+^) *m*/*z*: [*M*]^.+^: calcd for [C_77_H_104_N_8_S_2_Si_4_]^.+^: 1316.6897; found 1316.6911; correct isotope distribution. Single‐crystalline specimen were obtained by slow diffusion of methanol into a chloroform solution of **3 e**.

### OPV fabrication and characterization

Pre‐patterned indium tin oxide (ITO) substrates were ultrasonically cleaned for 8 min in acetone followed by 8 min in isopropanol. The substrates were then treated with an O_2_ plasma for 10 min. A cesium doped zinc oxide (ZnO:Cs) solution was spin‐coated on the substrates following a previously reported recipe.^24^, ^25^ The ZnO:Cs films were annealed at 300 °C for 30 min. The substrates were then transferred to a nitrogen filled glovebox. For the active layer **PTB7** (Purchased from 1‐Material Inc) and the acceptor molecules were separately dissolved in 10 mg mL^−1^ chlorobenzene adding 1 % 1,8‐diiodooctane. Donor and acceptors were mixed in a 1:1 volume ratio. The active layer was statically spin‐coated on the ZnO:Cs layer for 60 s at 800 rpm followed by 5 s at 2000 rpm and annealed at 80 °C for 10 min. The devices were completed by thermal evaporation of a 10 nm MoO_3_ layer and an 80 nm Ag anode. Both the external quantum efficiency (EQE) and the current–voltage characteristics of the BHJ solar cells were measured using a Keithley 2450 Source Measure Unit. The current–voltage characteristics were measured under simulated solar light from a Sun 3000 Solar Simulator (class AAA, Abet Technologies) under an AM 1.5G light illumination (100 mW cm^−2^). The light intensity was corrected by the mismatch factor using a silicon reference diode (VLSI Standards Inc.).

## Conflict of interest

The authors declare no conflict of interest.

## Supporting information

As a service to our authors and readers, this journal provides supporting information supplied by the authors. Such materials are peer reviewed and may be re‐organized for online delivery, but are not copy‐edited or typeset. Technical support issues arising from supporting information (other than missing files) should be addressed to the authors.

Supplementary
