# Supplementary material for: Azaacene Dimers: Acceptor Materials with a Twist
Source: Chemistry. 2019 Dec 19;26(2):412–8. doi: 10.1002/chem.201904683 (PMC6973103; doi:10.1002/chem.201904683)
Supplement: Supplementary file 1 — Supplementary [file CHEM-26-412-s001.pdf]

CHEMISTRY
A European Journal

Supporting Information

Azaacene Dimers: Acceptor Materials with a Twist

Lukas Ahrens,[a] Julian Butscher,[b] Victor Brosius,[a] Frank Rominger,[a] Jan Freudenberg,*[a] Yana Vaynzof,*[b] and Uwe H. F. Bunz*[a]

chem_201904683_sm_miscellaneous_information.pdf

Sections

S1. General remarks..S3
S2. Synthesis……..S6
S3. UV-Vis spectra...S11
S4. Electrochemistry/cyclovoltammetry...S12
S5. Quantum-chemical calculations S14
S6 Polarized light microscopy…………………………………………………………...S15
S7. NMR spectra…...S16
S8. X-ray single-crystal structure analysis...S21

S1. General Remarks

Reagents and solvents for synthesis

All reagents were obtained from commercial suppliers and were used without further purification. Deuterated solvents were purchased from Sigma-Aldrich Laborchemikalien GmbH (Seelze, Germany). All reactions requiring exclusion of oxygen and moisture were carried out in heat-gun dried glassware under a dry and oxygen free nitrogen or argon atmosphere using Schlenk and glovebox techniques.

Column chromatography

Column chromatography was performed using silica gel from Macherey, Nagel & Co. KG (Düren, Germany) (particle size: 0.040 - 0.063 mm). For TLC Polygram Sil G/UV 254 plates from Macherey, Nagel & Co. KG (Düren, Germany) were used and examined under UV-light irradiation (254 nm and 365 nm). Before column chromatography the crude product was mixed with Celite 545 and DCM to make a slurry. The solvent was removed by rotary evaporation to get a dry powder.

Gel permeation chromatography

Preparative GPC was performed on Bio-Beads® (S-X1 Beads, 200 – 400 Mesh, crosslinked polystyrene), purchased from Bio-Rad Laboratories, Inc., using toluene as solvent.

^{1}H NMR spectra and ^{13}C NMR spectra

All spectra were recorded at room temperature on a Bruker Avance III 600 (^{1}H: 600 MHz, ^{13}C: 151 MHz), Bruker Avance III 500 (^{1}H: 500 MHz, ^{13}C: 126 MHz) or Bruker Avance III 300 (^{1}H: 301 MHz). ^{13}C NMR spectra were measured proton decoupled if not stated otherwise. NMR spectra were integrated and processed using the Software TopSpin 3.5pl5 (Bruker). For calibration the residual solvent peaks were referenced.[1] Chemical shifts δ are reported in ppm and coupling constants J in Hz. The following abbreviations describe the observed multiplicities: s = singlet, d = doublet, m = multiplet.

IR spectra

All spectra were recorded neat at room temperature on a Jasco FT/IR-4100. Signals are reported in wavenumbers [cm^{-1}].

Mass spectrometry

The mass spectra were recorded using the following instruments: Bruker ApexQe hybrid 9.4 T FT-ICR (MALDI); Bruker AutoFlex Speed time-of-flight (MALDI).

Melting points

Melting points were determined in open glass capillaries with a Melting Point Apparatus MEL-TEMP (Electrothermal, Rochford, UK).

Cyclic voltammetry

The cyclic voltammetry (CV) experiments were carried out using PGSTAT101 Potentiostat Galvanostat with a platinum working electrode (Metrohm 6.120.4190, diameter 1 mm), a platinum wire auxiliary electrode (Metrohm 3.109.0790), a silver wire reference electrode (Metrohm 6.1241.060), a 0.1 mol L^{-1} NBu_4PF_6 solution in degassed, dry DCM, and ferrocene/ferrocenium as the reference redox system and internal standard (−5.1 eV) at room temperature and 0.2 V s^{-1}. To determine the reduction and oxidation potentials, the half-wave potentials were used.[2]

X-ray single-crystal structure analysis

X-ray single-crystal structure analyses were measured on a Bruker Smart APEX-II Quazar Area Detector diffractometer by Dr. F. Rominger (Heidelberg University). Diffraction intensities were corrected for Lorentz and polarization effects. An empirical absorption correction was applied using SADABS based on the Laue symmetry of reciprocal space. Heavy atom diffractions were solved by direct methods and refined against F2 with the full matrix least square algorithm. Hydrogen atoms were either isotropically refined or calculated. The structures were solved and refined using the SHELXTL software package.

UV-Vis and fluorescence spectroscopy

Absorption spectra were recorded on a Jasco UV-Vis V-660 or Jasco UV-Vis V-670. Fluorescence spectra were recorded on a Jasco FP-6500.

Quantum yields

Quantum yields were determined by an Ulbricht sphere (6 inch) using a PTI QuantaMaster 40 equipped with a Hamamatsu R928P Photomultiplier.

Quantum lifetimes

Quantum lifetimes were determined on a Horiba FluoroCube-01-NL lifetime spectrofluorometer with emission monochromator (Seya-Namioka type, 200 nm to 800 nm) and diode excitation (Nano-LED N-375L, 375 ± 10 nm, < 200 ps).

Camera

The photos were taken by a Canon EOS 7D under daylight or UV-light irradiation (λ = 365 nm).

Computational studies

Computational studies were carried out using DFT calculations on Turbomole 6.3.1 and Gaussian16. Geometry optimizations were performed using the B3LYP functional and def2-TZVP basis set. At this geometry, the absolute energy and FMO energies were assigned by a single-point approach at the B3LYP/6-311++G** level of theory.[3]

S2. Synthesis

3,6-Bis((tri*iso*propylsilyl)ethynyl)benzene-1,2-diamine (**2a**),[4] 1,4-bis((tri*iso*propylsilyl)ethynyl)-naphthalene-2,3-diamine (**2b**),[5] 1,4-bis((tri*iso*propylsilyl)ethynyl)anthracene-2,3-diamine (**2c**),[4] 1,4-bis((tri*iso*propylsilyl)ethynyl)phenazine-2,3-diamine (**2d**),[6] 4,7-bis((tri*iso*propylsilyl)-ethynyl)benzo[*c*][1,2,5]thiadiazole-5,6-diamine (**2e**),[7] 1,4-bis-((tri*iso*propylsilyl)ethynyl)-phenazine (**4a**),[8] 6,11-bis((tri*iso*propylsilyl)ethynyl)benzo[*b*]phenazine (**4b**),[5] 6,13-bis((tri*iso*-propylsilyl)ethynyl)naphtho[2,3-*b*]phenazine (**4c**)[9] and 6,13-bis((tri*iso*propylsilyl)-ethynyl)quinoxalino[2,3-*b*]phenazine (**4d**)[10] were synthesized according to literature procedures.

General procedure:

In a heatgun dried Schlenk tube under an atmosphere of argon was added quinone **1** (1.00 equiv.) and the *ortho*-diamine **2** (2.00 equiv). Then chloroform and acetic acid were added and the reaction mixture stirred at 50 °C for 15 h. The mixture was cooled to room temperature and diluted with water (10 mL). The phases were separated, and the aqueous layer was extracted with dichloromethane (3×10 mL). The combined organic phases were washed with sodium bicarbonate solution (10 mL), dried over magnesium sulfate and filtrated. The solvent was removed under reduced pressure and the crude product was absorbed on Celite®. After flash column chromatography (petroleum ether/diethylether 250:1 v/v) and gel permeation chromatography (toluene) the product **3** was isolated.

3,3,3',3'-Tetramethyl-2,2',3,3'-tetrahydro-1,1'-spirobi[1*H*-indene]-5,5',6,6'-tetraone (**1**)

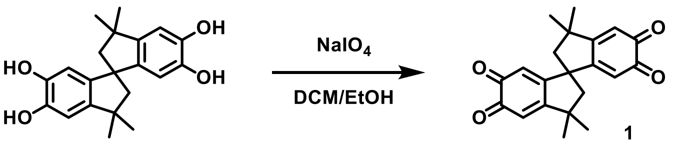

In a flask was dissolved racemic 3,3,3',3'-tetramethyl-2,2',3,3'-tetrahydro-1,1'-spirobi[1*H*-indene]-5,5',6,6'-tetraol (3.00 g, 8.81 mmol, 1.00 equiv.) in 40 mL ethanol and was then diluted with 150 mL dichloromethane. A solution of sodium periodate (4.15 g, 19.4 mmol, 2.20 equiv.) in 40 mL distilled water was added and the reaction mixture stirred at room temperature for 1 h. The phases were separated, and the aqueous layer was extracted with dichloromethane (3×30 mL). The combined organic phases were washed with brine (20 mL), dried over magnesium sulfate and filtrated. The solvent was removed under reduced pressure and the title compound **1** was isolated as a red solid (2.78 g, 8.28 mmol, 94 %).

^{1}H NMR ($CDCl_3$, 301 MHz, RT): δ = 6.27 (d, J = 0.55 Hz, 2H), 6.17 (d, J = 0.55 Hz, 2H), 2.38 (d, J = 13.5 Hz, 2H), 2.24 (d, J = 13.5 Hz, 2H), 1.41 (s, 6H), 1.39 (s, 6H) ppm. All analytical data is in good agreement with literature.[11] Single crystalline specimen was obtained by slow diffusion of methanol into a chloroform solution of **1**.

4,11-Bis((tri*iso*propylsilyl)ethynyl)-[1,2,5]thiadiazolo[3,4-*b*]phenazine (**4e**)

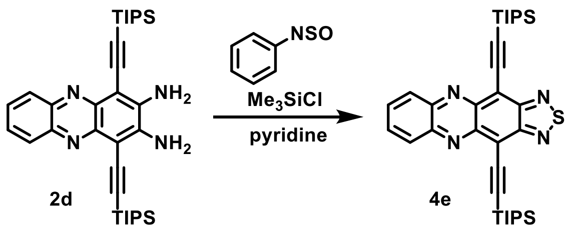

In a heatgun dried Schlenk tube under an atmosphere of argon was suspended **2d** (100 mg, 175 µmol, 1.00 equiv.) in 1.5 mL anhydrous pyridine. *N*-thionylaniline (41.3 µL, 51.2 mg, 368 µmol, 2.10 equiv.) was added and after stirring the reaction mixture at 80 °C for 5 min, trimethylsilyl chloride (222 µL, 190 mg, 1.75 mmol, 10.0 equiv.) was slowly added to the suspension. The reaction mixture was stirred at 80 °C for 6 h. The mixture was cooled to room temperature and diluted with 10 mL DCM. The mixture was washed with 1N hydrochloric acid (2 x 10 mL) and sodium bicarbonate solution (10 mL), dried over magnesium sulfate and filtrated. The solvent was removed under reduced pressure and the crude product was absorbed on Celite®. After flash column chromatography (petroleum ether/diethylether 250:1 v/v -> 100:1 -> 50:1) and gel permeation chromatography (toluene) the product **4e** was isolated as a dark blue solid (21.6 mg, 36.1 µmol, 21 %).

^{1}H NMR ($CDCl_3$, 301 MHz, RT): δ = 8.09 - 8.16 (m, 2H), 7.75 - 7.82 (m, 2H), 1.30 - 1.34 (m, 42H) ppm. All analytical data is in good agreement with literature.[12]

3,3,3',3'-Tetramethyl-6,6',9,9'-tetrakis((tri*iso*propylsilyl)ethynyl)-2,2',3,3'-tetrahydro-1,1'-spiro-bi[cyclopenta[*b*]phenazine] (**3a**)

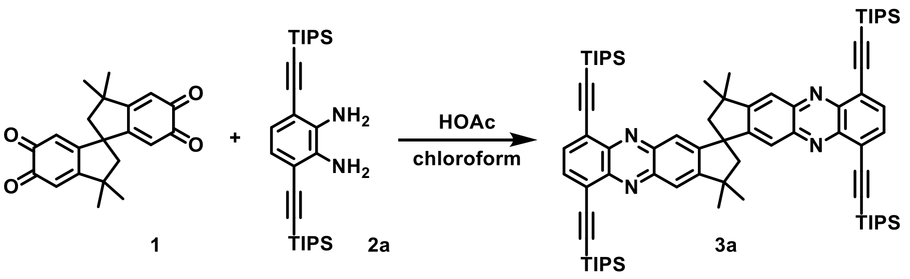

The **GP** was applied to **1** (100 mg, 297 µmol, 1.00 equiv.) and **2a** (279 mg, 595 µmol, 2.00 equiv.) in 1.5 mL chloroform and 1.5 mL acetic acid. Flash column chromatography (SiO_2; petroleum ether/diethyl ether 250:1 v/v) and gel permeation chromatography (toluene) yielded a yellow solid **3a** (237 mg, 197 µmol, 66%).

R_f = 0.76 (SiO_2; petroleum ether/dichloromethane 2:1, v/v). Mp: 185 °C. ^{1}H NMR ($CDCl_3$, 600 MHz, RT): δ = 8.06 (s, 2H), 7.90 (d, J = 7.40 Hz, 2H), 7.86 (d, J = 7.40 Hz, 2H), 7.64 (s, 2H), 2.66 - 2.73 (m, 4H), 1.70 (s, 6H), 1.60 (s, 6H), 1.27 - 1.31 (m, 42H), 1.06 - 1.11 (m, 42H) ppm. ^{13}C NMR ($CDCl_3$, 151 MHz, RT): δ = 158.2, 157.6, 143.8, 143.8, 143.0, 142.9, 133.6, 133.4, 124.9, 124.5, 124.2, 122.0, 104.1, 103.8, 100.8, 100.4, 60.3, 57.4, 44.0, 32.1, 30.6, 19.0, 18.8, 11.7, 11.5 ppm. IR (ATR): $\tilde{\nu}$ = 2941, 2863, 1461, 1036, 996, 880, 847, 787, 674, 660, 644, 633, 581, 457 cm^{-1}. HRMS (MALDI$^+$) m/z: $[M]^{\cdot+}$: calcd. for $[C_{77}H_{108}N_4Si_4]^{\cdot+}$: 1200.7646; found 1200.7693; correct isotope distribution.

3,3,3',3'-Tetramethyl-6,6',11,11'-tetrakis((tri*iso*propylsilyl)ethynyl)-2,2',3,3'-tetrahydro-1,1'-spirobi[benzo[*b*]cyclopenta[*i*]phenazine] (**3b**)

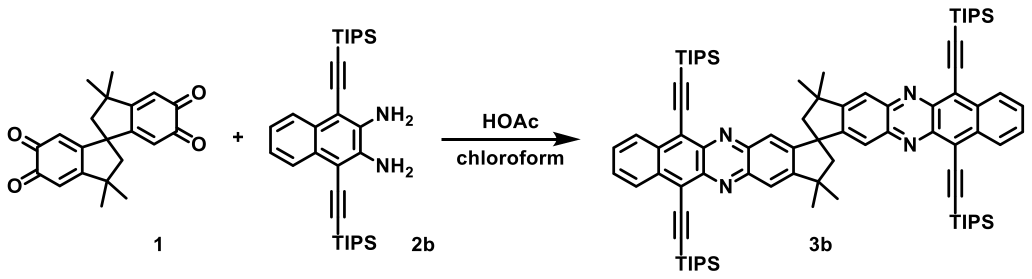

The **GP** was applied to **1** (40.0 mg, 119 µmol, 1.00 equiv.) and **2b** (123 mg, 238 µmol, 2.00 equiv.) in 1.0 mL chloroform and 1.0 mL acetic acid. Flash column chromatography (SiO_2; petroleum ether/diethyl ether 250:1 v/v) and gel permeation chromatography (toluene) yielded red solid **3b** (133 mg, 102 µmol, 86 %).

R_f = 0.74 (SiO_2; petroleum ether/dichloromethane 2:1, v/v). Mp: 212 °C. ^{1}H NMR ($CDCl_3$, 600 MHz, RT): δ = 8.75 (d, J = 8.08 Hz, 2H), 8.69 (d, J = 8.01 Hz, 2H), 8.06 (s, 2H), 7.68 (s, 2H), 7.59 - 7.67 (m, 4H), 2.66 - 2.80 (m, 4H), 1.74 (s, 6H), 1.64 (s, 6H), 1.33 - 1.44 (m, 42H), 1.09 - 1.21 (m, 42H) ppm. ^{13}C NMR ($CDCl_3$, 126 MHz, RT): δ = 158.8, 158.2, 144.9, 144.8, 141.1, 140.9, 135.2, 135.0, 127.9, 127.8, 127.7, 127.7, 125.0, 122.0, 120.9, 120.5, 108.2, 107.8, 103.2, 102.9, 60.2, 57.3, 43.9, 32.0, 30.6, 19.1, 18.9, 11.8, 11.6 ppm. IR (ATR): $\tilde{\nu}$ = 2941, 2862, 1461, 1442, 1389, 1367, 1042, 995, 879, 759, 675, 660, 593, 482, 403 cm^{-1}. HRMS (MALDI$^+$) m/z: $[M]^{\cdot+}$: calcd. for $[C_{85}H_{112}N_4Si_4]^{\cdot+}$: 1300.7959; found 1300.7983; correct isotope distribution.

3,3,3',3'-Tetramethyl-6,6',13,13'-tetrakis((tri*iso*propylsilyl)ethynyl)-2,2',3,3'-tetrahydro-1,1'-spirobi[cyclopenta[*b*]naphtho[2,3-*i*]phenazine] (**3c**)

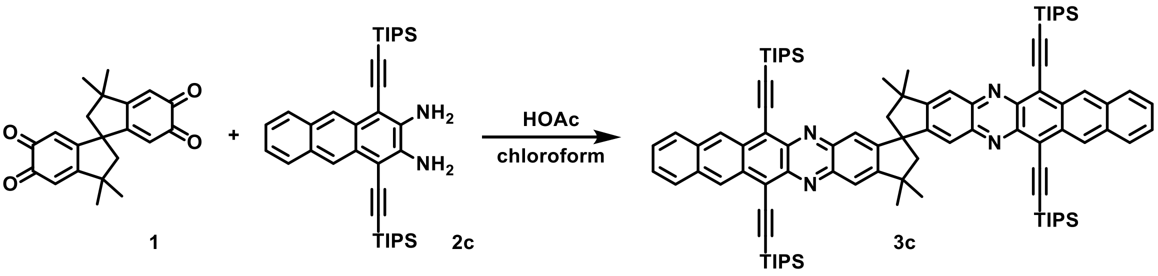

The **GP** was applied to **1** (85.7 mg, 255 µmol, 1.00 equiv.) and **2c** (290 mg, 510 µmol, 2.00 equiv.) in 1.5 mL chloroform and 1.5 mL acetic acid. Flash column chromatography (SiO_2; petroleum ether/diethyl ether 250:1 v/v) and gel permeation chromatography (toluene) yielded a green solid **3c** (251 mg, 179 µmol, 70%).

R_f = 0.73 (SiO_2; petroleum ether/dichloromethane 2:1, v/v). Mp: ≥400 °C. ^{1}H NMR ($CDCl_3$, 600 MHz, RT): δ = 9.42 (s, 2H), 9.37 (s, 2H), 8.00 (s, 2H), 7.97 - 8.03 (m, 4H), 7.64 (s, 2H), 7.43 - 8.48 (m, 4H), 2.75 (d, J = 13.5 Hz, 2H), 2.70 (d, J = 13.5 Hz, 2H), 1.74 (s, 6H), 1.64 (s, 6H), 1.40 - 1.48 (m, 42H), 1.16 - 1.26 (m, 42H) ppm. ^{13}C NMR ($CDCl_3$, 151 MHz, RT): δ = 159.1, 158.5, 145.3, 145.3, 140.9, 140.7, 133.0, 132.9, 132.7, 132.6, 128.8, 128.8, 127.0, 126.8, 126.8, 126.7, 125.2, 122.1, 120.8, 120.3, 109.6, 109.1, 104.2, 103.8, 60.1, 57.3, 44.0, 32.0, 30.5, 19.2, 19.0, 11.9, 11.7 ppm. IR (ATR): $\tilde{\nu}$ = 2940, 2862, 1460, 1375, 1139, 1016, 994, 751, 740, 668, 651, 578, 462, 418 cm^{-1}. HRMS (MALDI$^+$) m/z: [M]$^{\cdot+}$: calcd. for $[C_{93}H_{116}N_4Si_4]^{\cdot+}$: 1400.8272; found 1400.8243; correct isotope distribution.

3,3,3',3'-Tetramethyl-6,6',13,13'-tetrakis((tri*iso*propylsilyl)ethynyl)-2,2',3,3'-tetrahydro-1,1'-spirobi[cyclopenta[*b*]quinoxalino[2,3-*i*]phenazine] (**3d**)

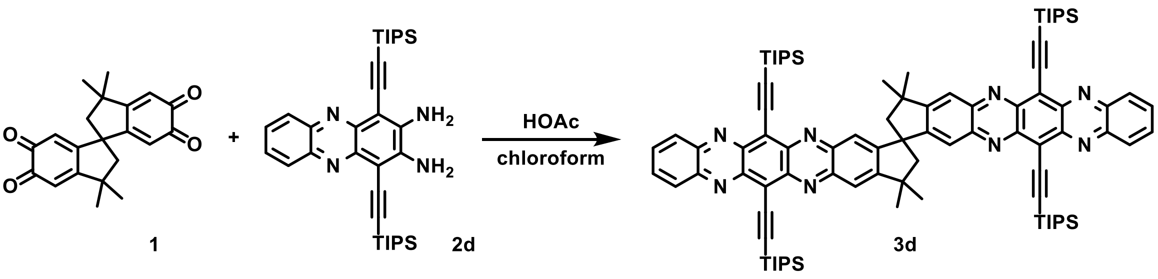

The **GP** was applied to **1** (100 mg, 297 µmol, 1.00 equiv.) and **2d** (279 mg, 5.95 µmol, 2.00 equiv.) in 1.5 mL chloroform and 1.5 mL acetic acid. Flash column chromatography (SiO_2; petroleum ether/diethyl ether 250:1 v/v -> 100:1 -> 50:1) and gel permeation chromatography (toluene) yielded green solid **3d** (277 mg, 197 µmol, 66 %).

R_f = 0.40 (SiO_2; petroleum ether/dichloromethane 2:1, v/v). Mp: ≥400 °C. ^1H NMR ($CDCl_3$, 500 MHz, RT): δ = 8.16 - 8.24 (m, 4H), 8.07 (s, 2H), 7.77 - 7.84 (m, 4H), 7.70 (s, 2H), 2.70 - 2.80 (m, 4H), 1.76 (s, 6H), 1.66 (s, 6H), 1.39 - 1.44 (m, 42H), 1.17 - 1.25 (m, 42H) ppm. ^{13}C NMR ($CDCl_3$, 126 MHz, RT): δ = 160.1, 159.4, 145.8, 145.8, 145.5, 145.5, 143.0, 142.9, 142.7, 142.5, 132.0, 132.0, 130.7, 130.6, 125.3, 122.9, 122.5, 122.4, 112.7, 112.1, 103.4, 103.1, 60.0, 57.5, 44.1, 32.0, 30.5, 19.2, 19.0, 11.9, 11.7 ppm. IR (ATR): $\tilde{\nu}$ = 2940, 2862, 1452, 1429, 1382, 1311, 1117, 1023, 996, 881, 734, 582, 419 cm^{-1}. HRMS (MALDI$^+$) m/z: $[M+H]^{\cdot+}$: calcd. for $[C_{89}H_{113}N_8Si_4]^{\cdot+}$: 1405.8160; found 1405.8148; correct isotope distribution.

3,3,3',3'-Tetramethyl-6,6',9,9'-tetrakis((tri*iso*propylsilyl)ethynyl)-2,2',3,3'-tetrahydro-1,1'-spiro-bi[cyclopenta[*b*]phenazine] (**3e**)

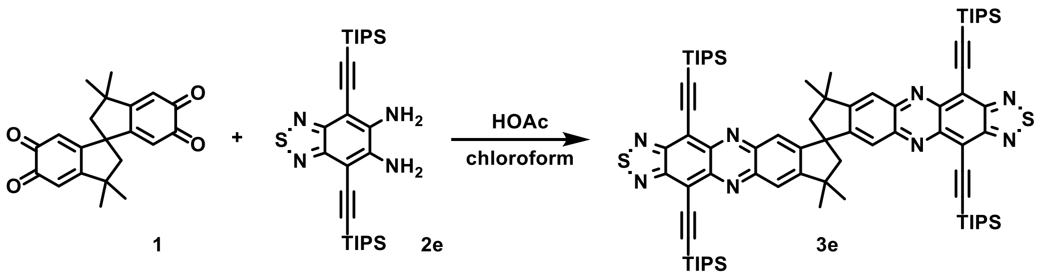

The **GP** was applied to **1** (150 mg, 446 µmol, 1.00 equiv.) and **2e** (470 mg, 892 µmol, 2.00 equiv.) in 2.0 mL chloroform and 2.0 mL acetic acid. Flash column chromatography (SiO_2; petroleum ether/diethyl ether 250:1 v/v -> 100:1 -> 50:1) and gel permeation chromatography (toluene) yielded green solid **3e** (389 mg, 295 µmol, 66 %).

R_f = 0.42 (SiO_2; petroleum ether/dichloromethane 2:1, v/v). Mp: 325 °C. ^1H NMR ($CDCl_3$, 600 MHz, RT): δ = 7.96 (s, 2H), 7.59 (s, 2H), 2.68 - 2.75 (m, 4H), 1.73 (s, 6H), 1.63 (s, 6H), 1.33 - 1.41 (m, 42H), 1.12 - 1.21 (m, 42H) ppm. ^{13}C NMR ($CDCl_3$, 126 MHz, RT): δ = 160.1, 159.5, 154.8, 154.7, 145.8, 145.7, 142.5, 142.4, 125.0, 122.2, 114.4, 114.1, 111.9, 111.4, 102.3, 102.1, 59.9, 57.3, 44.1, 31.9, 30.4, 19.1, 18.9, 11.8, 11.6 ppm. IR (ATR): $\tilde{\nu}$ = 2941, 2862, 1454, 1447, 1364, 1200, 1018, 996, 920, 861, 658, 646, 599, 572 cm^{-1}. HRMS (MALDI$^+$) m/z: $[M]^{\cdot+}$: calcd. for $[C_{77}H_{104}N_8S_2Si_4]^{\cdot+}$: 1316.6897; found 1316.6911; correct isotope distribution. Single crystalline specimen were obtained by slow diffusion of methanol into a chloroform solution of **3e**.

S3. UV-Vis spectra

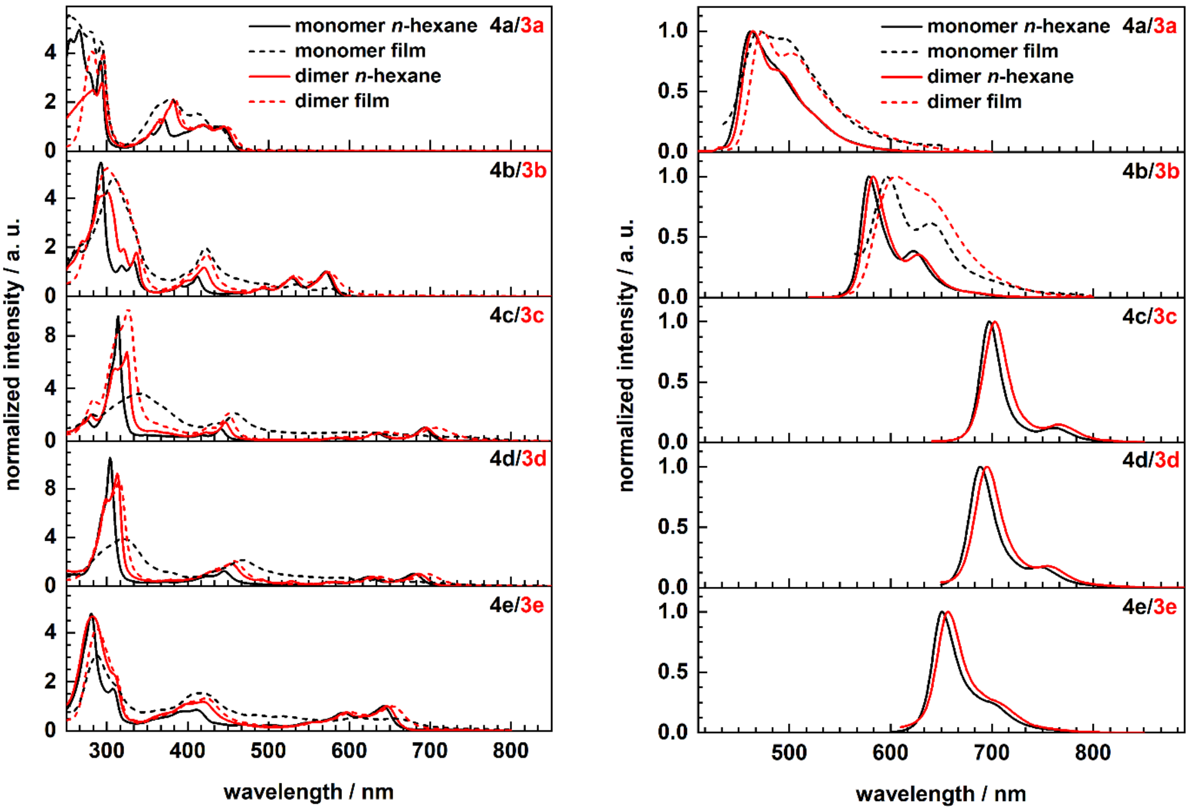

Figure S1: Normalized absorption (left) and emission (right) spectra of **3a-e** (red) and **4a-e** (black) in *n*-hexane (dilute solution) and thin films on glass (dashed; chloroform, 10 mg mL^{-1}). **3c-e** are non-fluorescent in thin films.

S4. Electrochemistry/cyclovoltammetry

The cyclic voltammetry (CV) experiments were carried out using a platinum working electrode, a platinum wire auxiliary electrode, a silver wire reference electrode, a 0.1 mol L^{-1} NBu_4PF_6 solution in degassed, dry DCM, and ferrocene/ferrocenium as the reference redox system and internal standard (−5.1 eV) at room temperature and 0.2 V s^{-1}. To determine the first reduction potentials ($E_{(0/-)}$) of **3a-e** and the first oxidation potential of ferrocene, the half-wave potentials were used.[2]

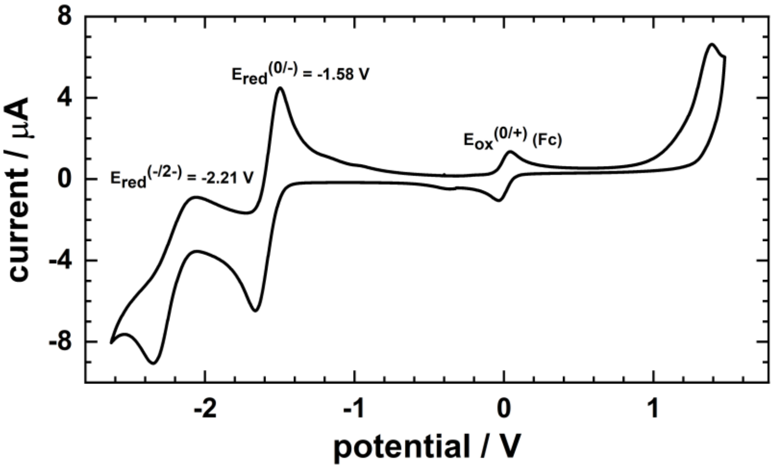

Figure S2: CV spectrum of 3,3,3',3'-tetramethyl-6,6',9,9'-tetrakis((tri*iso*propylsilyl)ethynyl)-2,2',3,3'-tetrahydro-1,1'-spirobi[cyclopenta[*b*]phenazine] (**3a**).

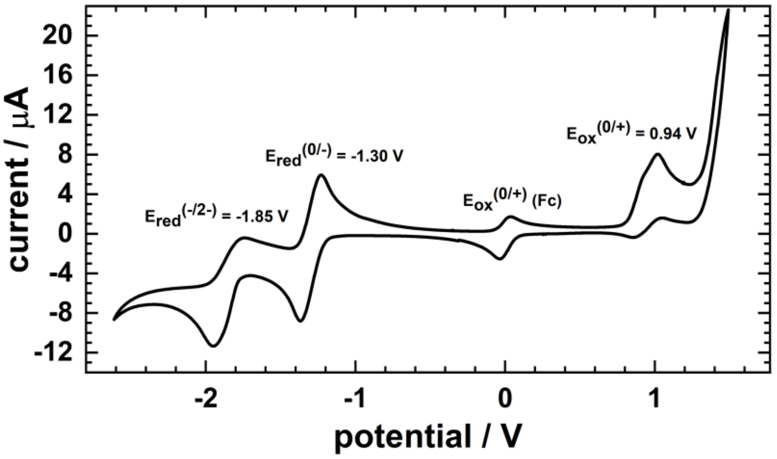

Figure S3: CV spectrum of 3,3,3',3'-Tetramethyl-6,6',11,11'-tetrakis((tri*iso*propylsilyl)ethynyl)-2,2',3,3'-tetrahydro-1,1'-spirobi[benzo[*b*]cyclopenta[*i*]phenazine] (**3b**).

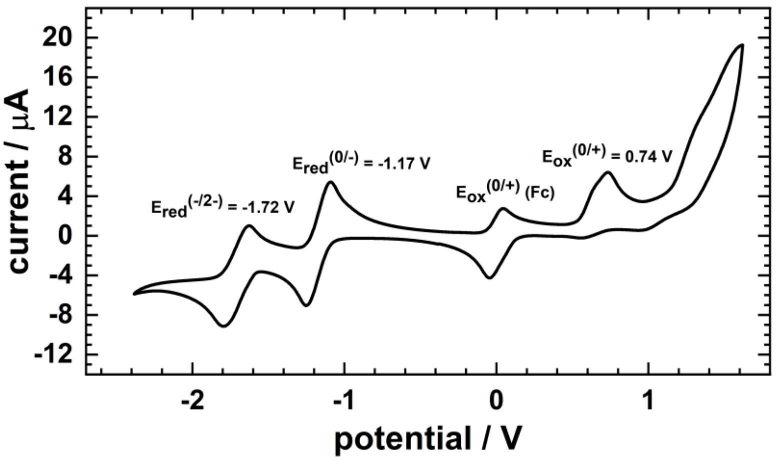

Figure S4: CV spectrum of 3,3,3',3'-tetramethyl-6,6',13,13'-tetrakis((tri*iso*propylsilyl)ethynyl)-2,2',3,3'-tetrahydro-1,1'-spirobi[cyclopenta[*b*]naphtho[2,3-*i*]phenazine] (**3c**).

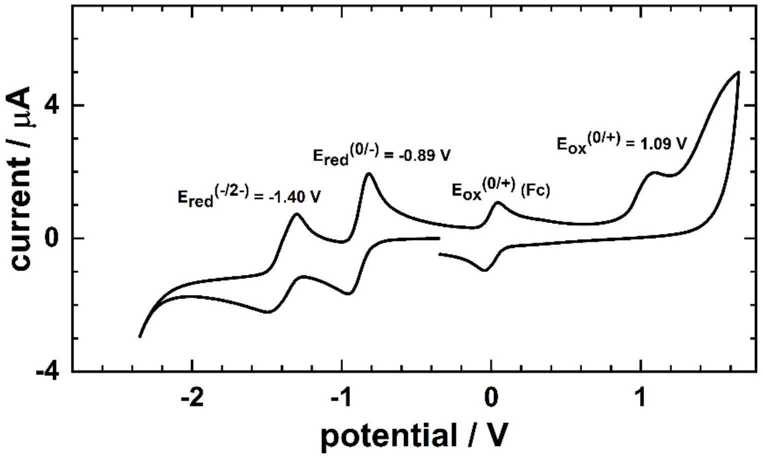

Figure S5: CV spectrum of 3,3,3',3'-tetramethyl-6,6',13,13'-tetrakis((tri*iso*propylsilyl)ethynyl)-2,2',3,3'-tetrahydro-1,1'-spirobi[cyclopenta[*b*]quinoxalino[2,3-*i*]phenazine] (**3d**).

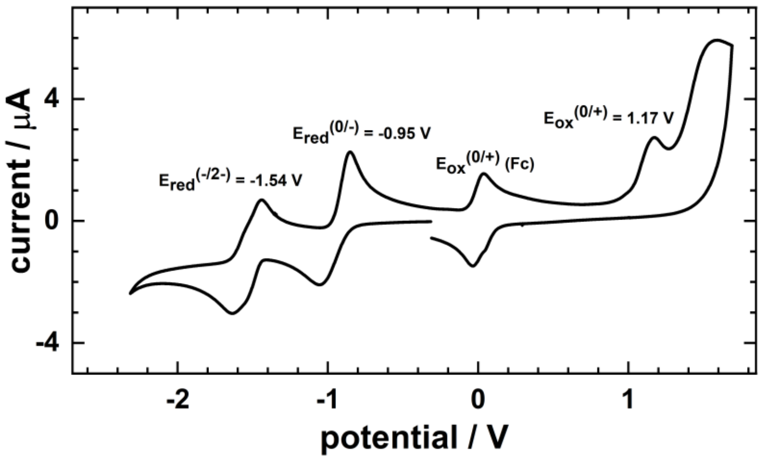

Figure S6: CV spectrum of 3,3,3',3'-tetramethyl-6,6',9,9'-tetrakis((tri*iso*propylsilyl)ethynyl)-2,2',3,3'-tetrahydro-1,1'-spirobi[cyclopenta[*b*]phenazine] (**3e**).

S5. Quantum-chemical calculations

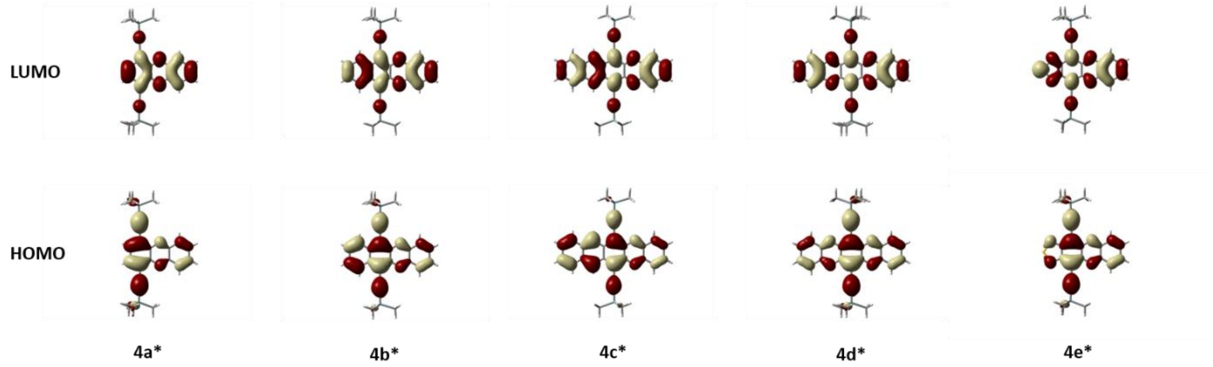

Figure S7: Quantum chemical calculations (TURBOMOLE B3LYP/def2-TZVP//Gaussian09 B3LYP/6-311++G**) of the FMOs (LUMOs top, HOMOs bottom) for compounds **4a*-e***. TMS groups were used instead of TIPS.

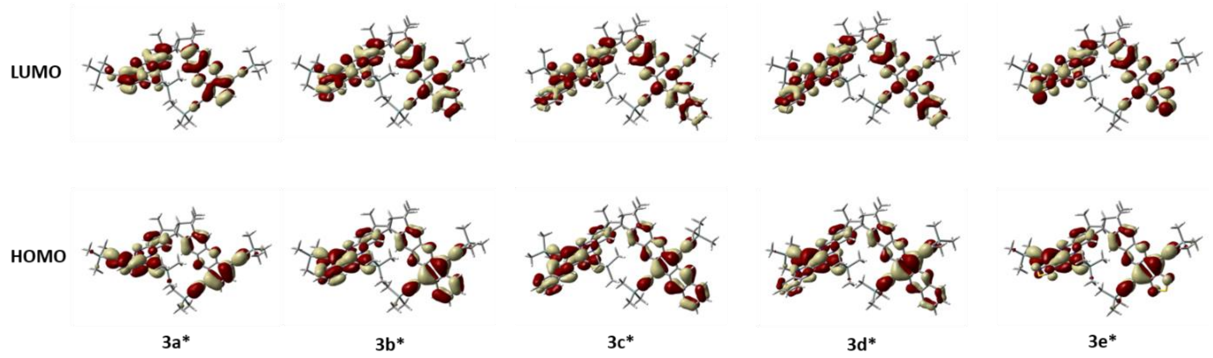

Figure S8: Quantum chemical calculations (TURBOMOLE B3LYP/def2-TZVP//Gaussian09 B3LYP/6-311++G**) of the FMOs (LUMOs top, HOMOs bottom) for compounds **3a*-e***. TMS groups were used instead of TIPS.

S6. Polarized light microscopy

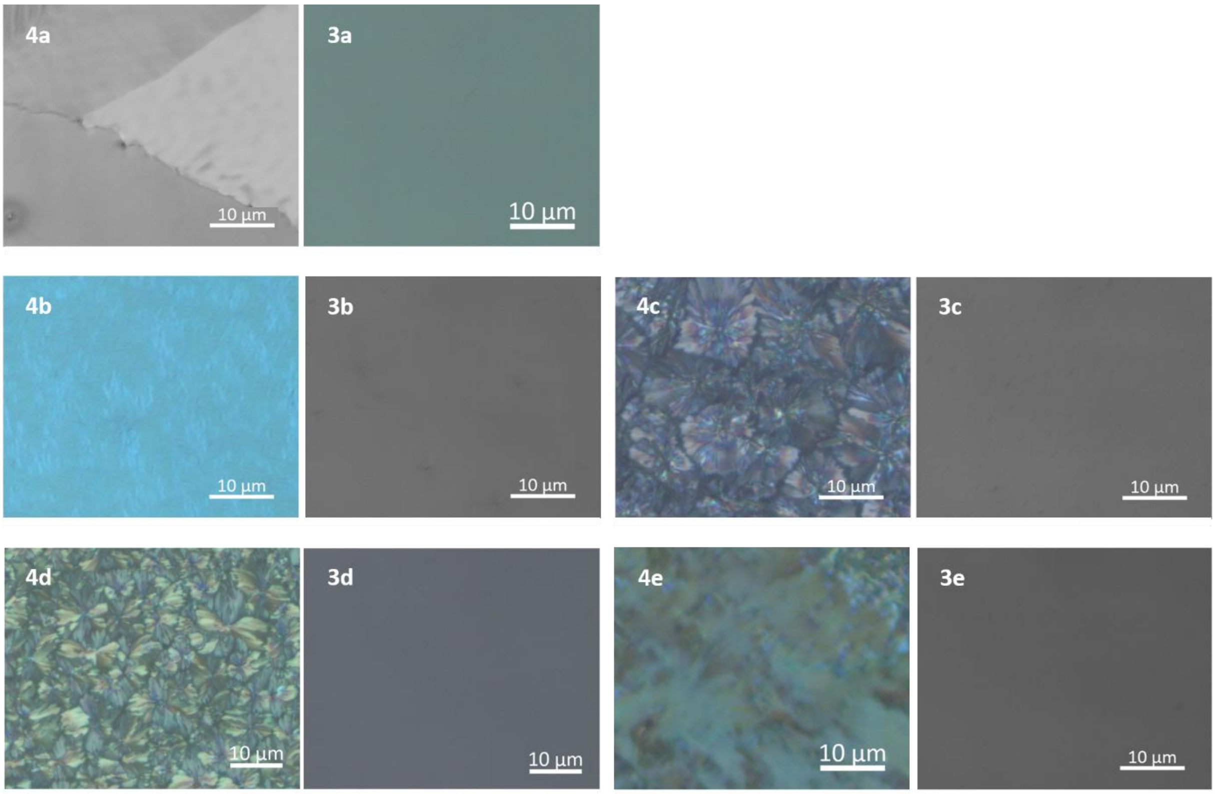

Figure 9. Microscopic images of spin-coated thin films of **4a-e** and **3a-e** on glass (chloroform, 10 mg mL^{-1}) under crossed polarizers.

S7. NMR spectra

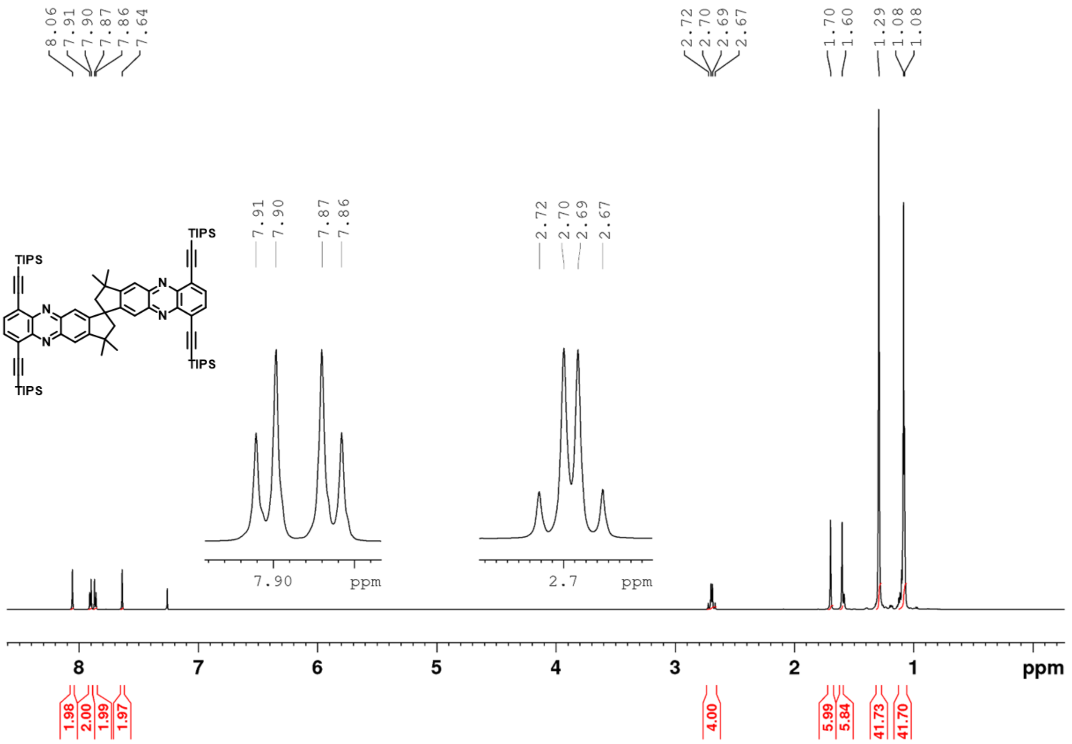

Figure S10: ^{1}H NMR spectrum (600 MHz) of **3a** in $CDCl_3$.

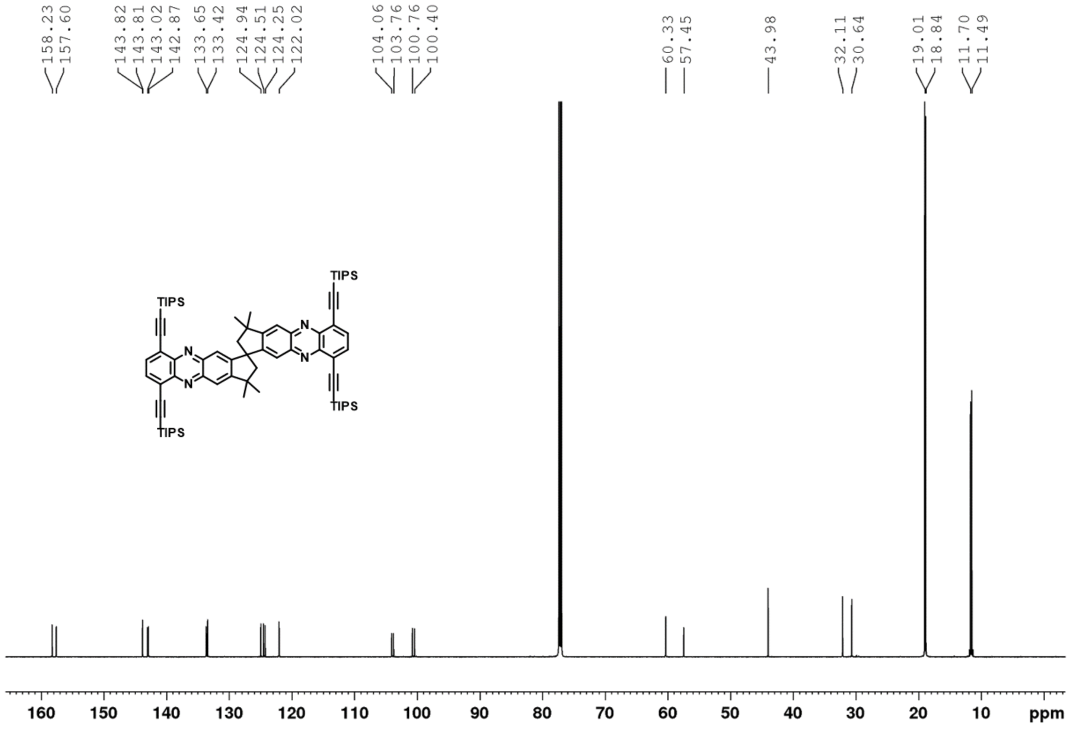

Figure S11: $^{13}C\{^{1}H\}$ NMR spectrum (151 MHz) of **3a** in $CDCl_3$.

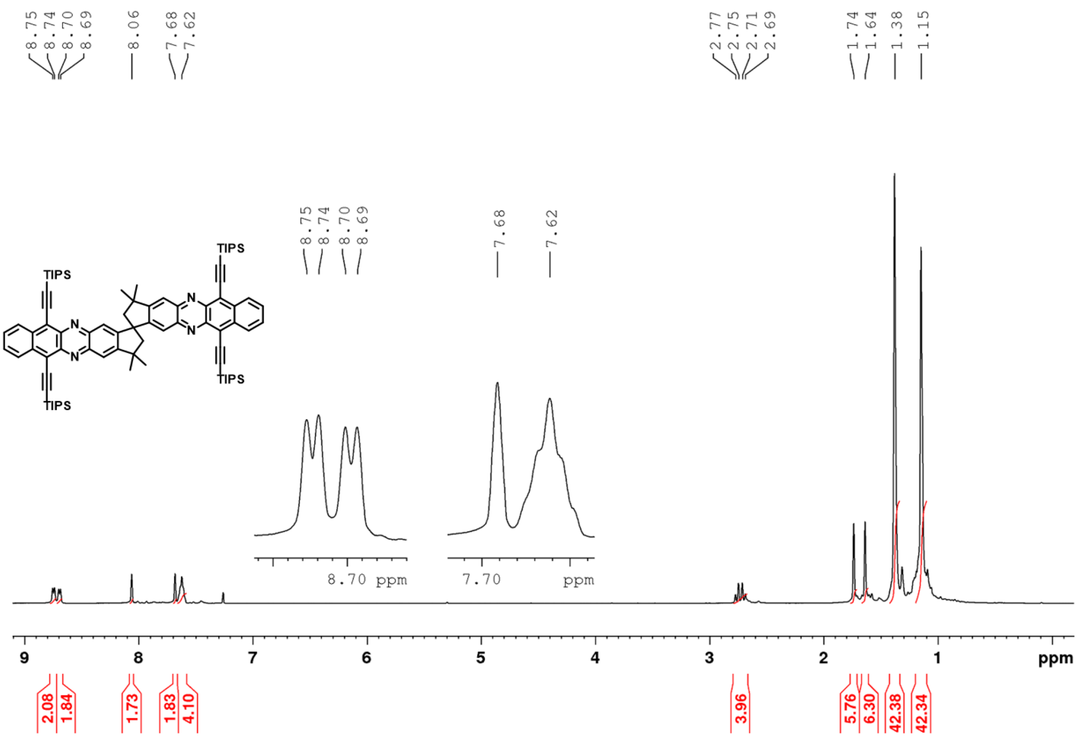

Figure S12: ^{1}H NMR spectrum (500 MHz) of **3b** in $CDCl_3$.

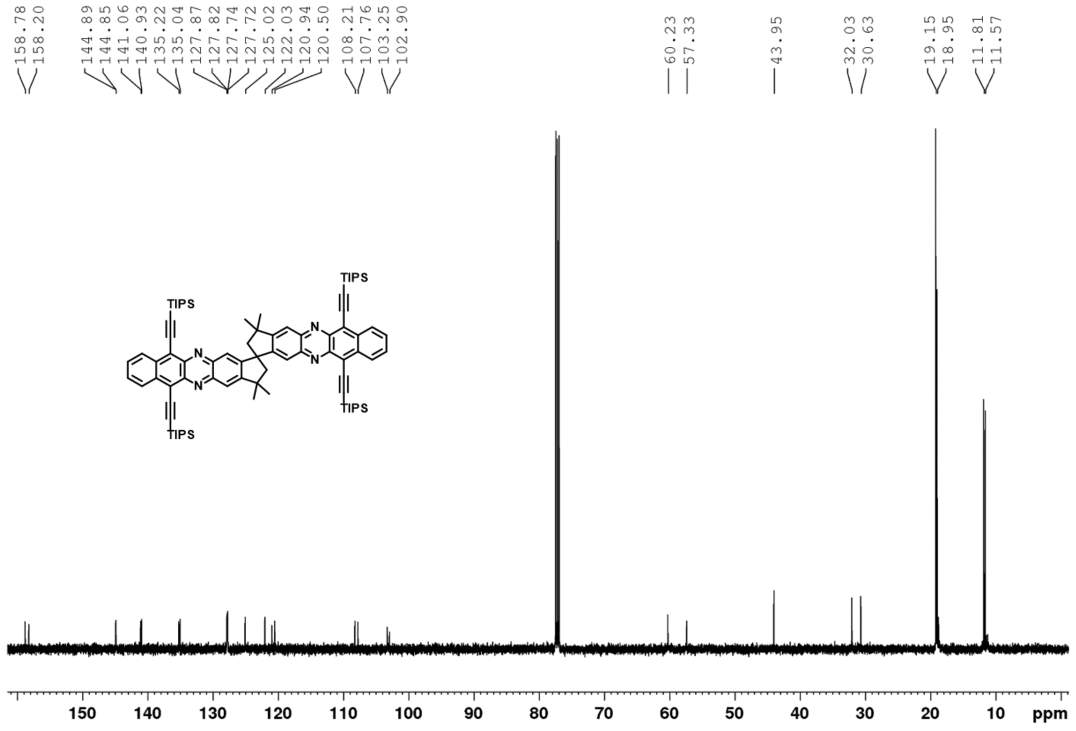

Figure S13: $^{13}C\{^{1}H\}$ NMR spectrum (126 MHz) of **3b** in $CDCl_3$.

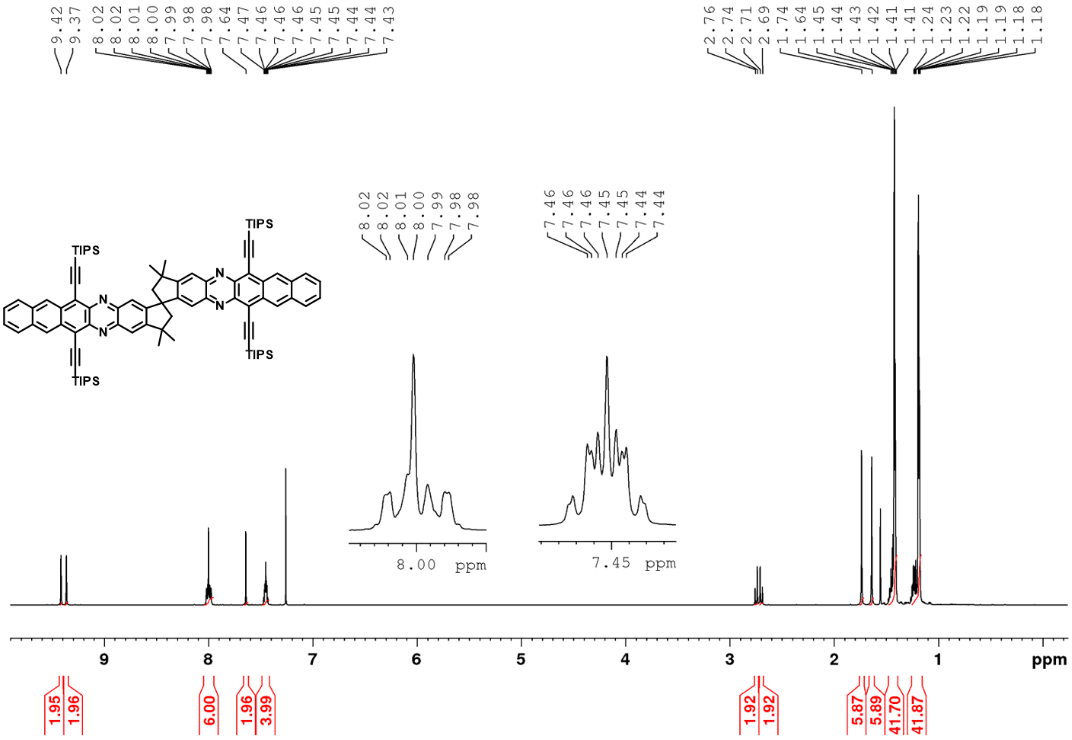

Figure S14: ^{1}H NMR spectrum (600 MHz) of **3c** in $CDCl_3$.

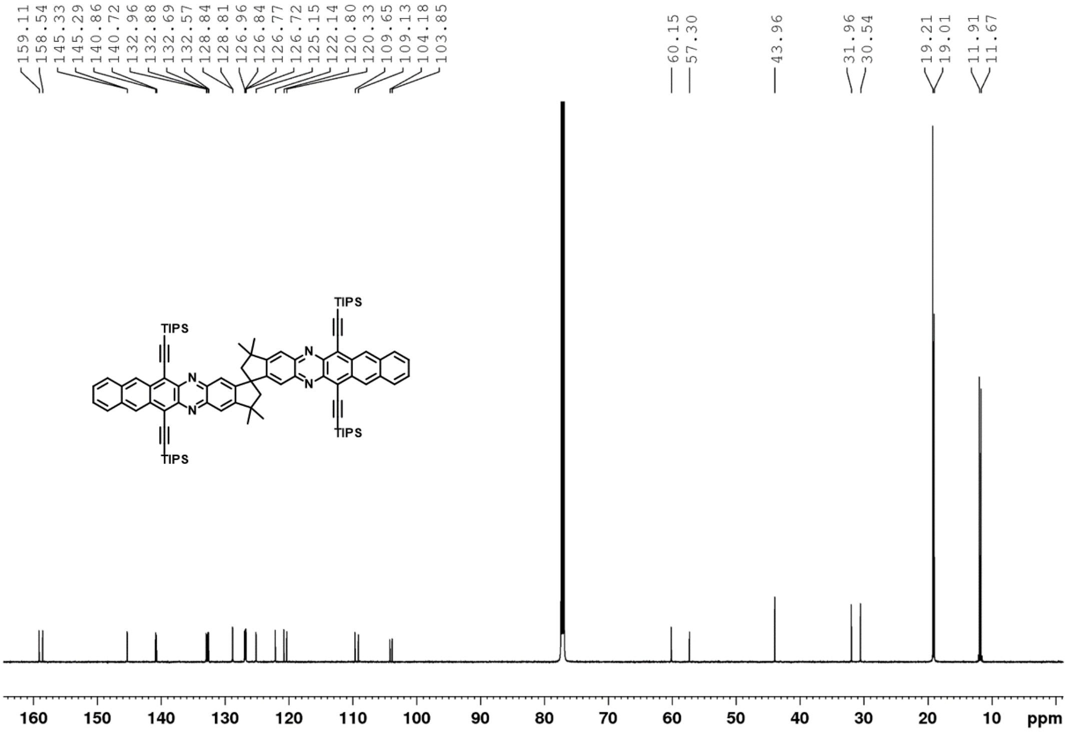

Figure S15: ^{13}C{^{1}H} NMR spectrum (151 MHz) of **3c** in $CDCl_3$.

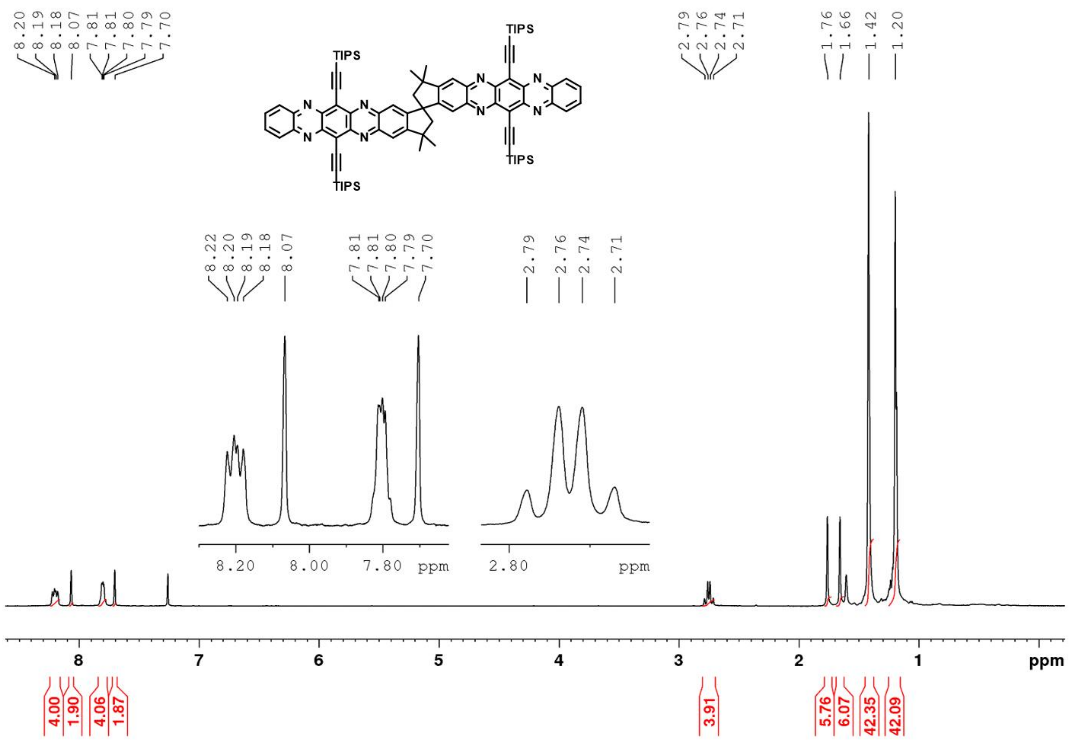

Figure S16: ^{1}H NMR spectrum (500 MHz) of **3d** in $CDCl_3$.

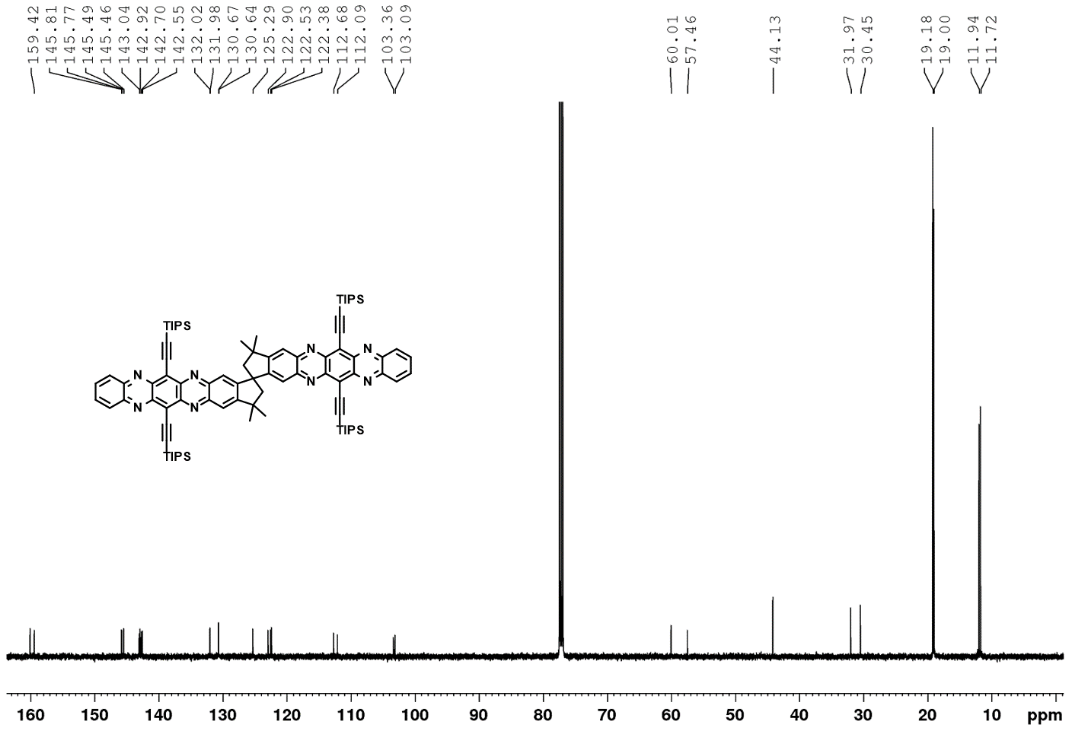

Figure S17: $^{13}C\{^{1}H\}$ NMR spectrum (126 MHz) of **3d** in $CDCl_3$.

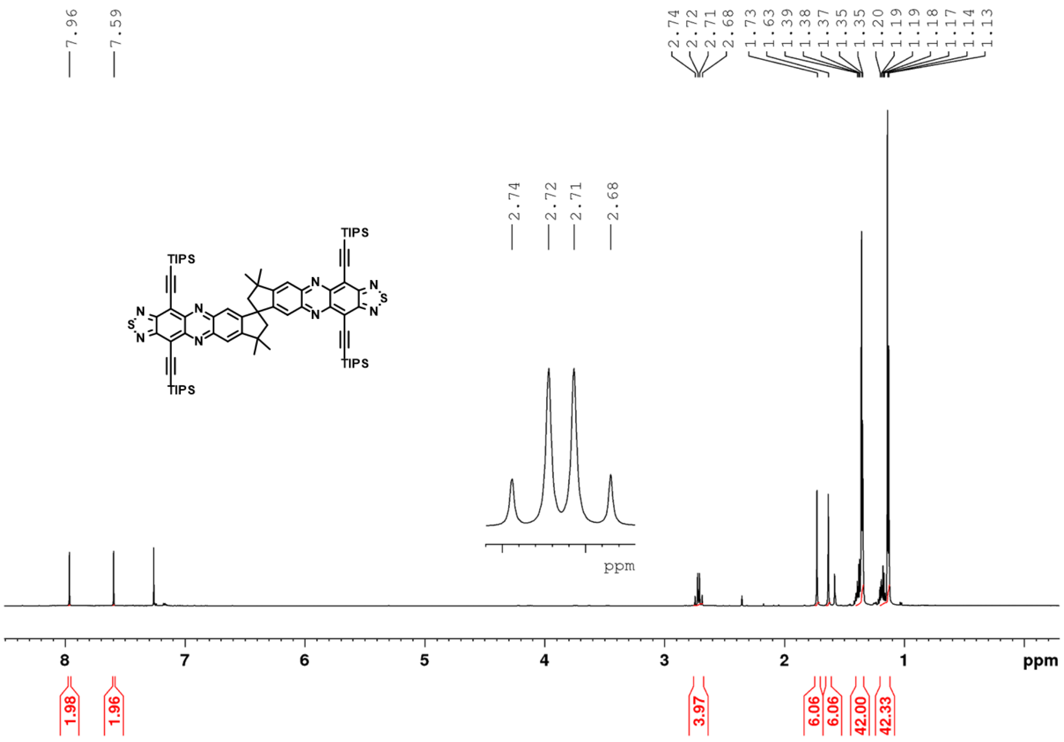

Figure S18: ^{1}H NMR spectrum (600 MHz) of **3e** in $CDCl_3$.

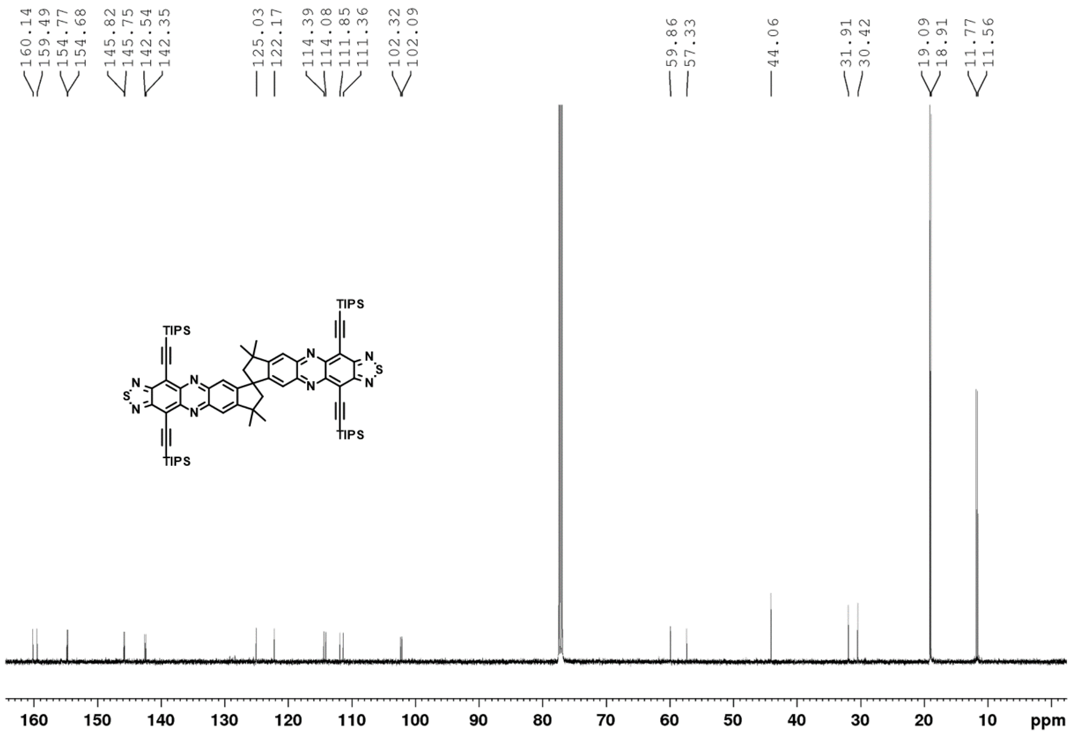

Figure S19: ^{13}C{^{1}H} NMR spectrum (126 MHz) of **3e** in $CDCl_3$.

S8. X-ray single-crystal structure analysis

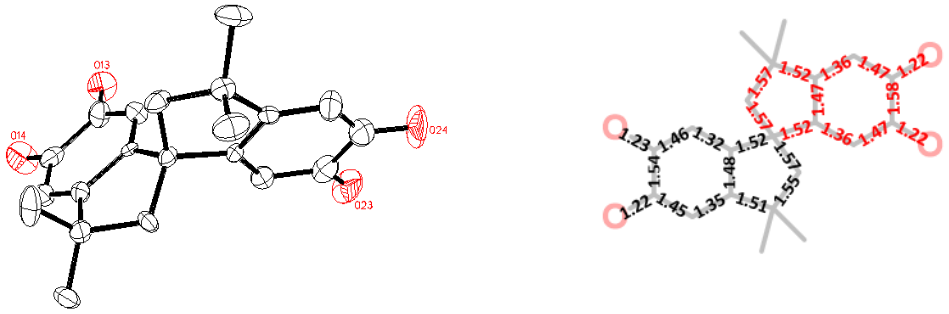

Figure S20*:* Left: ORTEP structure of **1** (CCDC 1958581); right: bond lengths, measured in black, calculated (TURBOMOLE B3LYP/def2-TZVP) in red.

Empirical formula	$C_{21}H_{20}O_4$
Formula weight	336.37
Temperature	200(2) K
Wavelength	0.71073 Å
Crystal system	tetragonal
Space group	$P4_1$
Z	8
Unit cell dimensions	a = 10.7698(13) Å α = 90 deg.
	b = 10.7698(13) Å β = 90 deg.
	c = 29.980(4) Å γ = 90 deg.
Volume	3477.4(10) $Å^3$
Density (calculated)	1.28 g/cm^3
Absorption coefficient	0.09 mm^{-1}
Crystal shape	brick
Crystal size	0.127 x 0.118 x 0.031 mm^3
Crystal colour	orange
Theta range for data collection	1.9 to 23.5 deg.
Index ranges	$-12 \leq h \leq 11$, $-12 \leq k \leq 12$, $-33 \leq l \leq 33$

Reflections collected	18481
Independent reflections	5124 (R(int) = 0.0990)
Observed reflections	3435 (I > 2σ(I))
Absorption correction	Semi-empirical from equivalents
Max. and min. transmission	0.96 and 0.74
Refinement method	Full-matrix least-squares on F^2
Data/restraints/parameters	5124 / 1 / 459
Goodness-of-fit on F^2	1.04
Final R indices (I>2sigma(I))	R1 = 0.068, wR2 = 0.168
Absolute structure parameter	0.1(10)
Largest diff. peak and hole	0.38 and -0.26 eÅ^{-3}

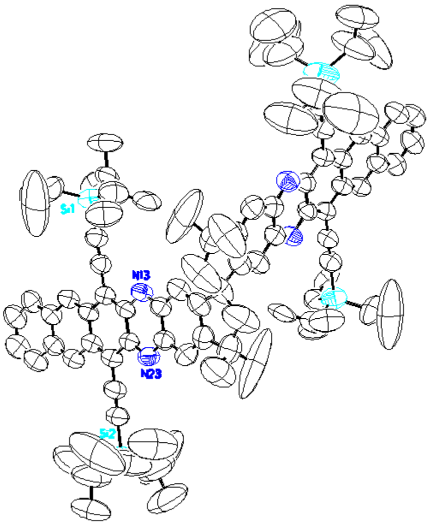

Figure S21*:* ORTEP structure of **3c** (CCDC 1958582).

Empirical formula	$C_{93}H_{116}N_4Si_4$
Formula weight	1402.25
Temperature	200(2) K
Wavelength	1.54178 Å
Crystal system	orthorhombic

Space group	Pccn
Z	4
Unit cell dimensions	a = 17.2322(13) Å α = 90 deg.
	b = 33.906(4) Å β = 90 deg.
	c = 14.9653(12) Å γ = 90 deg.
Volume	8743.7(14) Å^3
Density (calculated)	1.07 g/cm^3
Absorption coefficient	0.96 mm^{-1}
Crystal shape	plank
Crystal size	0.142 x 0.085 x 0.025 mm^3
Crystal colour	green
Theta range for data collection	4.1 to 47.8 deg.
Index ranges	$-16 \le h \le 13$, $-32 \le k \le 32$, $-14 \le l \le 14$
Reflections collected	30793
Independent reflections	4043 (R(int) = 0.1269)
Observed reflections	2420 ($I > 2\sigma(I)$)
Absorption correction	Semi-empirical from equivalents
Max. and min. transmission	1.48 and 0.77
Refinement method	Full-matrix least-squares on F^2
Data/restraints/parameters	4043 / 973 / 538
Goodness-of-fit on F^2	1.12
Final R indices (I>2sigma(I))	R1 = 0.119, wR2 = 0.273
Largest diff. peak and hole	0.35 and -0.32 eÅ^{-3}

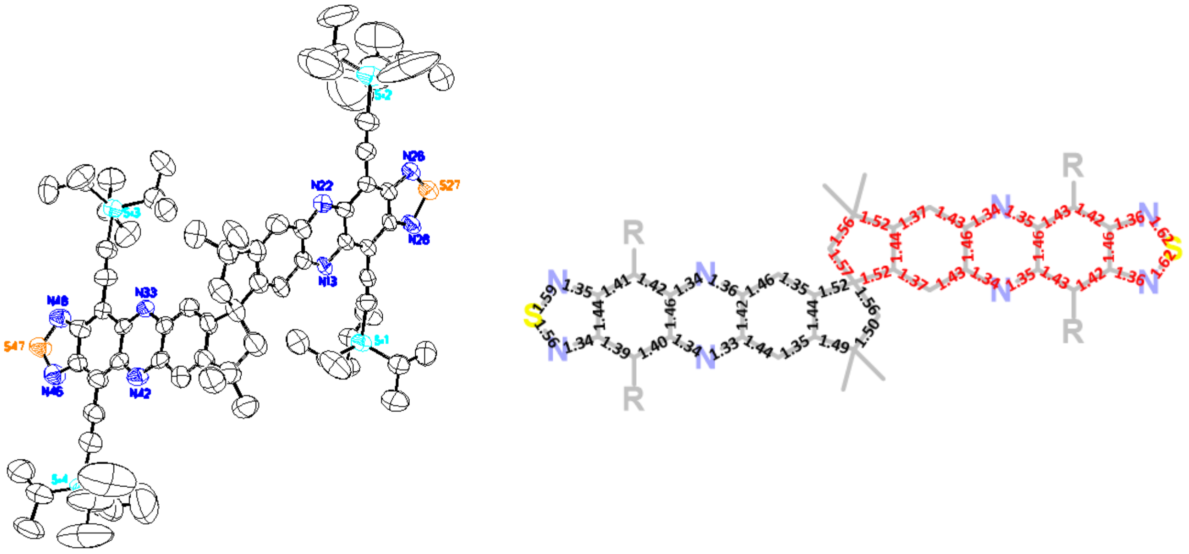

Figure S22*:* Left: ORTEP structure of **3e** (CCDC 1958583); right: bond lengths, measured in black, calculated (TURBOMOLE B3LYP/def2-TZVP) in red.

Empirical formula	$C_{77}H_{104}N_8S_2Si_4$
Formula weight	1318.16
Temperature	200(2) K
Wavelength	1.54178 Å
Crystal system	orthorhombic
Space group	$P_{na}2_1$
Z	4
Unit cell dimensions	a = 14.9932(7) Å α = 90 deg.
	b = 16.6654(7) Å β = 90 deg.
	c = 30.2092(19) Å γ = 90 deg.
Volume	7548.3(7) $Å^3$
Density (calculated)	1.16 g/cm^3
Absorption coefficient	1.60 mm^{-1}
Crystal shape	plank
Crystal size	0.175 x 0.070 x 0.017 mm^3

Crystal colour	green
Theta range for data collection	3.0 to 48.5 deg.
Index ranges	$-14 \le h \le 14$, $-16 \le k \le 10$, $-29 \le l \le 29$
Reflections collected	29080
Independent reflections	7041 (R(int) = 0.1502)
Observed reflections	3818 ($I > 2\sigma(I)$)
Absorption correction	Semi-empirical from equivalents
Max. and min. transmission	1.46 and 0.78
Refinement method	Full-matrix least-squares on F^2
Data/restraints/parameters	7041 / 1801 / 821
Goodness-of-fit on F^2	1.02
Final R indices (I>2sigma(I))	R1 = 0.082, wR2 = 0.176
Absolute structure parameter	0.41(6)
Largest diff. peak and hole	0.60 and -0.37 eÅ^{-3}

[1] G. R. Fulmer, A. J. Miller, N. H. Sherden, H. E. Gottlieb, A. Nudelman, B. M. Stoltz, J. E. Bercaw, K. I. Goldberg, *Organometallics* **2010,** *29*, 2176-2179.

[2] C. M. Cardona, W. Li, A. E. Kaifer, D. Stockdale, G. C. Bazan, *Adv. Mater.* **2011**, *23*, 2367-2371.

[3] Gaussian 09, Revision A.02, M. J. Frisch, G. W. Trucks, H. B. Schlegel, G. E. Scuseria, M. A. Robb, J. R. Cheeseman, G. Scalmani, V. Barone, B. Mennucci, G. A. Petersson, H. Nakatsuji, M. Caricato, X. Li, H. P. Hratchian, A. F. Izmaylov, J. Bloino, G. Zheng, J. L. Sonnenberg, M. Hada, M. Ehara, K. Toyota, R. Fukuda, J. Hasegawa, M. Ishida, T. Nakajima, Y. Honda, O. Kitao, H. Nakai, T. Vreven, J. A. Montgomery, Jr., J. E. Peralta, F. Ogliaro, M. Bearpark, J. J. Heyd, E. Brothers, K. N. Kudin, V. N. Staroverov, R. Kobayashi, J. Normand, K. Raghavachari, A. Rendell, J. C. Burant, S. S. Iyengar, J. Tomasi, M. Cossi, N. Rega, J. M. Millam, M. Klene, J. E. Knox, J. B. Cross, V. Bakken, C. Adamo, J. Jaramillo, R. Gomperts, R. E. Stratmann, O. Yazyev, A. J. Austin, R. Cammi, C. Pomelli, J. W. Ochterski, R. L. Martin, K. Morokuma, V. G. Zakrzewski, G. A. Voth, P. Salvador, J. J. Dannenberg, S. Dapprich, A. D. Daniels, Ö. Farkas, J. B. Foresman, J. V. Ortiz, J. Cioslowski, D. J. Fox, Gaussian, Inc. Wallingford CT, **2009**.

[4] B. D. Lindner, B. A. Coombs, M. Schaffroth, J. U. Engelhart, O. Tverskoy, F. Rominger, M. Hamburger, U. H. F. Bunz, *Org. Lett.* **2013**, *15*, 666-669.

[5] S. Miao, S. M. Brombosz, P. v. R. Schleyer, J. I. Wu, S. Barlow, S. R. Marder, K. I. Hardcastle, U. H. F. Bunz, *J. Am. Chem. Soc.* **2008**, *130*, 7339-7344.

[6] B. D. Lindner, J. U. Engelhart, O. Tverskoy, A. L. Appleton, F. Rominger, A. Peters, H. Himmel, U. H. F. Bunz, *Angew. Chem. Int. Ed.* **2011**, *50*, 8588-8591.

[7] C. An, S. Zhou, M. Baumgarten, *Cryst. Growth Des.* **2015**, *154*, 1934-1938.

[8] J. J. Bryant, Y. Zhang, B. D. Lindner, E. A. Davey, A. L. Appleton, X. Qian, U. H. F. Bunz, J. Org. Chem. **2012**, *77*, 7479-7486.

[9] A. L. Appleton, S. M. Brombosz, S. Barlow, J. S. Sears, J.-L. Bredas, S. R. Marder, U. H. F. Bunz, *Nat. Commun*. **2010**, *1*, 1-6.

[10] S. Miao, A. L. Appleton, N. Berger, S. Barlow, S. Marder, K. Hardcastle, U. H. F. Bunz, *Chem. Eur. J.* **2009**, *15*, 4990-4993.

[11] B. S. Ghanem, N. B. McKeown, P. M. Budd, D. Fritsch, *Macromolecules* **2008**, *41*, 1640-1646.

[12] B. D. Lindner, F. Paulus, A. L. Appleton, M. Schaffroth, J. U. Engelhart, K. M. Schelkle, O. Tverskoy, F. Rominger, M. Hamburger, U. H. F. Bunz, *J. Mater. Chem. C* **2014**, *2*, 9609-9612.
